# *Lymnaea schirazensis*, an Overlooked Snail Distorting Fascioliasis Data: Genotype, Phenotype, Ecology, Worldwide Spread, Susceptibility, Applicability

**DOI:** 10.1371/journal.pone.0024567

**Published:** 2011-09-29

**Authors:** María Dolores Bargues, Patricio Artigas, Messaoud Khoubbane, Rosmary Flores, Peter Glöer, Raúl Rojas-García, Keyhan Ashrafi, Gerhard Falkner, Santiago Mas-Coma

**Affiliations:** 1 Departamento de Parasitología, Facultad de Farmacia, Universidad de Valencia, Valencia, Spain; 2 Münchner Malakologische Mitteilungen Heldia, Friedrich-Held Gesellschaft, Hetlingen, Germany; 3 Laboratorio de Biología de Parásitos y Vectores, Escuela de Biología, Benemérita Universidad Autónoma de Puebla, Puebla, México; 4 Department of Medical Microbiology and Parasitology, School of Medicine, Gilan University of Medical Sciences, Rasht, Iran; 5 Staatliches Museum für Naturkunde Stuttgart, Stuttgart, Germany; 6 Département Systématique et Evolution, UMS “Taxonomie et Collections”, Muséum National d’Histoire Naturelle, Paris, France; Universidade Federal de Minas Gerais, Brazil

## Abstract

**Background:**

Lymnaeid snails transmit medical and veterinary important trematodiases, mainly fascioliasis. Vector specificity of fasciolid parasites defines disease distribution and characteristics. Different lymnaeid species appear linked to different transmission and epidemiological patterns. Pronounced susceptibility differences to absolute resistance have been described among lymnaeid populations. When assessing disease characteristics in different endemic areas, unexpected results were obtained in studies on lymnaeid susceptibility to *Fasciola*. We undertook studies to understand this disease transmission heterogeneity.

**Methodology/Principal Findings:**

A ten-year study in Iran, Egypt, Spain, the Dominican Republic, Mexico, Venezuela, Ecuador and Peru, demonstrated that such heterogeneity is not due to susceptibility differences, but to a hitherto overlooked cryptic species, *Lymnaea schirazensis*, confused with the main vector *Galba truncatula* and/or other *Galba*/*Fossaria* vectors. Nuclear rDNA and mtDNA sequences and phylogenetic reconstruction highlighted an old evolutionary divergence from other *Galba*/*Fossaria* species, and a low intraspecific variability suggesting a recent spread from one geographical source. Morphometry, anatomy and egg cluster analyses allowed for phenotypic differentiation. Selfing, egg laying, and habitat characteristics indicated a migration capacity by passive transport. Studies showed that it is not a vector species (n = 8572 field collected, 20 populations): snail finding and penetration by *F. hepatica* miracidium occur but never lead to cercarial production (n = 338 experimentally infected).

**Conclusions/Significance:**

This species has been distorting fasciolid specificity/susceptibility and fascioliasis geographical distribution data. Hence, a large body of literature on *G. truncatula* should be revised. Its existence has henceforth to be considered in research. Genetic data on livestock, archeology and history along the 10,000-year post-domestication period explain its wide spread from the Neolithic Fertile Crescent. It is an efficient biomarker for the follow-up of livestock movements, a crucial aspect in fascioliasis emergence. It offers an outstanding laboratory model for genetic studies on susceptibility/resistance in *F. hepatica*/lymnaeid interaction, a field of applied research with disease control perspectives.

## Introduction

Freshwater snails of the family Lymnaeidae (Gastropoda) act as intermediate hosts or vectors of numerous digenean trematode species. Many lymnaeid species are of applied interest as they transmit several trematode species of large-scale medical and veterinary impact, among which fasciolids are the most important [1]. *Fasciola hepatica* and *F. gigantica* are two large-sized fasciolid trematode parasite species that cause fascioliasis, a disease which affects humans and livestock species almost everywhere [2]. This highly pathogenic liver parasitosis has been emerging in many countries of Latin America, Europe, Africa and Asia in the last two decades [2], [3]. This emergence phenomenon has partly been related to climate change [4], [5], given the high dependence of both lymnaeid snails and fasciolid larval stages on climatic and environmental characteristics [6]–[8].

Although livestock species play an important reservoir role, transmission studies have shown that the metacercarial infective stage from different origins, such as sheep, cattle, pig and donkey, represent similar infectivity sources [9], [10]. On the contrary, the specificity of fasciolid species regarding concrete lymnaeid species [1] represent a crucial factor in establishing the geographical distribution of the disease in both animals and humans. Moreover, disease prevalences and intensities also depend on the ecological characteristics (population dynamics, anthropophylic characteristics, type of water bodies, etc.) of the different lymnaeid vector species. That is why different lymnaeid species appear linked to the different transmission patterns and epidemiological scenarios of this very heterogeneous disease in humans [11], [12]. The continental differences in lymnaeid faunas also explain that in the Americas fascioliasis is only caused by *F. hepatica*, owing to the absence of lymnaeids of the genus *Radix* which act as transmitters of *F. gigantica* [1]. Likewise, as in other vector-borne diseases, this relationship supports the use of lymnaeids as biomarkers of the disease at both local and large scales and can thus be useful for the validation of mathematical modelling and remote sensing–geographical information system (RS-GIS) tools for the control of the disease [8], [13].

Despite the applied interest of lymnaeid snails, the present knowledge on the genetics of this gastropod group as well as on their parasite-host interrelationships is far from being sufficient. A good example of this situation is the systematic-taxonomic confusion in which this molluscan family has been immersed [1]. At lymnaeid species level, the problems are found mainly due to the interspecific morphological and anatomic uniformity numerous species present, usually resulting in serious difficulties in specimen classification, sometimes even impeding it [14]–[16]. Moreover, intraspecific variation of shell shape is particularly well marked within lymnaeids depending on environmental conditions [17], [18], although a genetic component in shell shape has been shown at least in some lymnaeid populations [19]. Thus, there are many specimen classification problems, mainly related to: (i) species of the “stagnicoline” group in Europe and North America [14], [20]; (ii) the “radix” group in Europe and Asia [20]; (iii) the “fossarine” or “*Galba*/*Fossaria*” group in the Americas [15] (*Fossaria* is a synonym of *Galba* [21]; terms “fossarine” or “*Galba*/*Fossaria*” group here used only in the meaning frequently found in American malacological literature of the last century).

At snail host level, trematodes show a marked specificity, from usually oioxenous (one digenean species/one snail species) or stenoxenous (one digenean species/a few, closely related snail species, i.e. those belonging to the same genus) to less frequently oligoxenous (one digenean species/numerous, family-, subfamily- or tribe-related snail species) [22]–[24]. Variability in the susceptibility of a concrete snail species to infection by a concrete digenean species has shown to be related to differences between snail populations and also between individuals among a concrete snail population [24], [25]. Differences in compatibility between a trematode species and different geographical populations of the same snail host species are known, including *Fasciola* [26], [27]. Among lymnaeids, pronounced differences in susceptibility have been highlighted among snail populations encountered in close proximity [28], [29], and some snail populations have been mentioned to even show a total lack of susceptibility or resistance [29]–[31].

However, the aforementioned different susceptibility phenomena in lymnaeids have to be considered with great caution. Many of these lymnaeid species involved in fascioliasis transmission maintain a confusing systematic-taxonomic status. When comparing different lymnaeid DNA sequences, several populations originally classified as belonging to different species showed identical DNA marker sequences, and other populations originally classified as pertaining to the same species presented different DNA marker sequences. Sometimes sequence differences were very few, suggesting intraspecific variability (different haplotypes). However, occasionally differences detected among populations classified as pertaining to the same species were numerous, sufficient as to consider different species involved. Moreover, the number of sequence differences between species sometimes appeared lower than that between populations of the same species [1], [14], [16]. This clearly underlines both the classification problems and the systematic-taxonomic confusion present in Lymnaeidae. Consequently, several susceptibility differences described could in fact be related to different lymnaeid species instead of different populations of the same lymnaeid species.

The crucial implications of lymnaeid vectors for fascioliasis transmission, epidemiology and control demonstrate the importance of developing new tools to facilitate specimen classification, genetic characterisation of natural populations and laboratory strains, and to elucidate the systematics and taxonomy of Lymnaeidae. The failure of all malacological and non-malacological tools applied to date suggests the analysis of DNA sequences and phylogenetic methods to be worthwhile. The first attempt made by a research collaboration of parasitologists, molecularists and malacologists was successful [1]. This success, together with the rapid realisation that locally restricted studies were insufficient because of the very large geographical distribution and spreading capacities of many lymnaeids belonging to very confusing lymnaeid groups, suggested early on that large, transboundary studies would be needed. In this sense, a worldwide lymnaeid molecular characterisation initiative was instigated [12], [16]. The great spreading capacity of lymnaeids means that sometimes not even the continental scale is sufficient, and intercontinental sequence comparisons are needed to classify specimens correctly. As an example: the sympatric *Lymnaea viatrix* and *L. cubensis*, which were noted to be involved in the human fascioliasis high hyperendemic area of the Bolivian Altiplano [32], later proved to only involve morphologically variable *Galba truncatula* of European origin [33]–[35]. The intercontinental spreading of lymnaeids and its role in fascioliasis dissemination is well known [2], [27], [36].

Of the different DNA markers used hitherto in lymnaeids, the 18S gene of the nuclear ribosomal DNA (rDNA) appears to be too conserved and its few variable positions may only be useful at generic and suprageneric taxon levels [15], [16], [33], [37]. Ribosomal DNA ITS-2 and secondarily ITS-1 are the most useful sequences for studies at species level [1], [14], [15], [35], [38], [39], [40]. These two spacers are useful for: (i) classification of lymnaeid specimens, (ii) characterisation of lymnaeid intraspecific genetic interpopulational variablity to furnish the genetic base on which to understand fasciolid-lymnaeid specificity, different susceptibilities or compatibilities of geographical strains or even resistances, (iii) establishment of valid species and their geographical distributions, and (iv) assessment of species interrelationships to arrange a natural systematic-taxonomic classification, which will allow for an analysis of coevolution with fasciolids [12], [16]. Interestingly, one mutation at the level of the ITS-1 and another at ITS-2 have proved useful to distinguish between resistant and susceptible populations of *P. columella* in Cuba [41], although nothing evidently suggests that these mutations are linked to resistance/susceptibility.

Within mitochondrial DNA (mtDNA), only fragments of 16S and *cox*1 have been sequenced in lymnaeids [15], [40], [42]–[44]. Recent knowledge indicates that mtDNA markers, including both mitochondrial genes and the ribosomal 12S and 16S genes within the mitochondrial genome, should be used with great caution when dealing with lymnaeid species belonging to different genera and even those well-separated within the same genus [45]. Of particular concern is the saturation of nucleotide positions. Additionally, it has also been documented that incomplete gene sequences do not necessarily contain a sufficiently significant portion of the whole gene, i.e. parts of the gene presenting evolutionary hot spots may be missed [46]. Consequently, the use of mtDNA markers for this initiative is restricted to (i) sequence comparisons and phylogenetic analyses of only closely related species within the same genus, (ii) studies of intraspecific variability of species by sequence comparisons of individuals and populations, (iii) genetic characterisation of laboratory strains, (iv) studies on the spread of populations of a species, and (v) studies on genetic exchange between different neighboring populations [12].

The present paper summarises results obtained in analyses of lymnaeid specimens of given populations originally ascribed to the main fascioliasis vector species *G. truncatula* in Europe, Asia, and Africa, and/or also to similar lymnaeid vector species belonging to the *Galba*/*Fossaria* group in North America, the Caribbean and South America. Such analyses were performed after obtaining unexpected results in experimental studies about lymnaeid susceptibility to *Fasciola* infection when assessing disease transmission characteristics in human hypo- to hyperendemic fascioliasis areas in the different continents. Multidisciplinary studies carried out to understand experimentally-tested, abnormal fasciolid susceptibility of these widely spread lymnaeid *G. truncatula*-like populations have taken more than 10 years. Characterisation studies have been made by nuclear rDNA and mtDNA sequences, phylogenetic tree reconstruction, phenotypic differentiation by shell morphometry, morphoanatomical characterisation, fecundation studies, ecological observations, and assessment of the geographical spread in correlation with historical events. These results demonstrate that another lymnaeid species, *Lymnaea* (*s. l.*) *schirazensis*, genetically distant but phenotypically very close, has always been confused with *G. truncatula* and/or other similar lymnaeid vector species in all these continents. The implications for fascioliasis are discussed, as this hitherto overlooked species has been distorting results of fasciolid specificity/susceptibility analyses as well as the geographical distribution of the disease. The existence of this *G. truncatula*-like lymnaeid species frequently present in animal fascioliasis endemic areas and usual in human fascioliasis endemic areas ought be henceforth considered to avoid misunderstandings concerning transmission. Moreover, results indicate that *L. schirazensis* can be used as a useful biomarker of foreign livestock introduction, a crucial aspect in fascioliasis spreading and emergence [12]. Additionally, *L. schirazensis* offers an outstanding new laboratory model for studies on genomics and proteomics about susceptibility/resistance in *F. hepatica*/lymnaeid interaction, an important field of applied research with disease control perspectives.

## Materials and Methods

### Lymnaeid snail material

**Figure 1:**
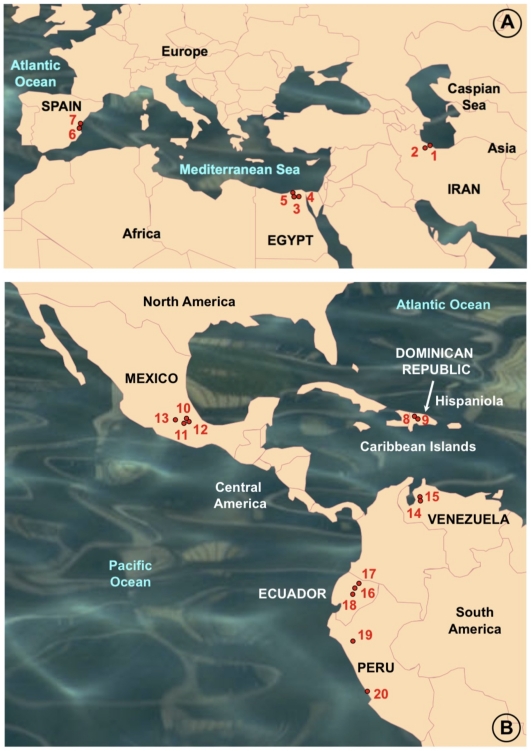
Maps of the Old and New Worlds showing localities where *Lymnaea schirazensis* was collected: A) Old World: 1 = Taleb Abad river, Bandar Anzali, Gilan province, Iran; 2 = Medicine Faculty, Rasht, Gilan province, Iran; 3 = El Kazza, Hosh Esa district, Behera governorate, Egypt; 4 = Tiba, Delengate district, Behera governorate, Egypt; 5 = Boulin El Aly, Kafr El Dawar district, Behera governorate, Egypt; 6 = Albufera of Valencia, Valencia province, Spain; 7 = Nules-Moncofar, Castellon province, Spain; B) New World: 8 = Constanza, Departamento de La Vega, the Dominican Republic; 9 = Río Grande, Constanza, Departamento de La Vega, the Dominican Republic; 10 = Los Molinos, subcuenca Nexapa, Atlixco, Puebla, Mexico; 11 = Escuela A. Obrego, La Trinidad Tepango, Atlixco, Puebla, Mexico; 12 = Xalpatlaco, Atlixco, Puebla, Mexico; 13 = Jiutepec, Morelos, Huauchinango, Mexico; 14 = Laguna de Fe y Alegria, El Valle, Estado de Merida, Venezuela; 15 = Hotel Valle Grande, El Valle, Estado de Merida, Venezuela; 16 = Guarandauco, Chillogallo, Ecuador; 17 = La Buena Esperanza, Cayambe, Ecuador; 18 = Machachi, Santo Domingo, Ecuador; 19 = Baños del Inca, Cajamarca, Peru; 20 = Rio Lurin, Lima, Peru. For a higher resolution situation of each locality, see respective coordenates in text.

The snail specimens studied (n = 8572 specimens) were collected in the field along a ten-year period, from 20 lymnaeid populations present in geographical areas with human and/or animal fascioliasis endemicity of eight countries (Figure 1):

Iran: 1) Taleb Abad river, Bandar Anzali, Gilan province (37°27′46″ N; 49°37′07″ E; −23 m below sea level-b.s.l.): 243 living specimens collected and analysed to assess potential natural infection by *Fasciola* through cercarial shedding verification; 2) garden of the Medicine Faculty, Rasht, Gilan province (37°11′39″ N; 49°38′04″ E; 26 m above sea level-a.s.l.): 81.
Egypt: 3) El Kazza, Hosh Esa district, Behera governorate (30°50′54″ N; 30°16′16″ E; 1 m a.s.l.): 1956; 4) Tiba, Delengate district, Behera governorate (30°50′34″ N; 30°29′11″ E; 4 m a.s.l.): 887; 5) Boulin El Aly, Kafr El Dawar district, Behera governorate (31°10′42″ N; 30°10′54″ E; 0 m a.s.l.): 369.
Spain: 6) Albufera of Valencia, Valencia province (39°17′42′′N; 0°20′41′′ W; −1 m b.s.l.): 1291; 7) Nules-Moncofar, Castellon province (39°50′01″ N; 0°06′28″ W; 34 m a.s.l.): 2077.
the Dominican Republic: 8) Constanza, Departamento de La Vega (18°54′25′′ N; 70°44 25′′ W; 1,184 m a.s.l): 265; 9) Río Grande, Constanza, Departamento de La Vega (18°52′37′′ N; 70°43′38′′ W; 1,182 m a.s.l.): 32.
Mexico: 10) Los Molinos, subcuenca Nexapa, Atlixco, Puebla (18°56′57″N; 98°23′25″W; 1,952 m a.s.l.): 149; 11) Escuela A. Obrego, La Trinidad Tepango, Atlixco, Puebla (18°51′46″ N; 98°26′33″ W; 1,**774 **m a.s.l.): 213; 12) Xalpatlaco, Atlixco, Puebla (18°55′58″ N; 98°26′22″ W; 1,903 m a.s.l.): 56; 13) Jiutepec, Morelos, Huauchinango (18°53′01″ N; 99°11′12″ W; 1,377 m a.s.l.): 31.
Venezuela: 14) Laguna de Fe y Alegría, El Valle, Estado de Merida (8°37′29″ N; 71°08′30″ W; 1,837 m a.s.l.): 38; 15) Hotel Valle Grande, El Valle, Estado de Merida (8°40′28″ N; 71°06′03″ W; 2,174 m a.s.l.): 114.
Ecuador: 16) Guarandauco, Chillogallo (0°17′48″ S; 78°38′55″ W; 3,158 m a.s.l.): 75; 17) La Buena Esperanza, Cayambe (0°03′39″ N; 78°08′25″ W; 2,821 m a.s.l.): 283; 18) Machachi, Santo Domingo (0°27′23″ S; 78°33′43″ W; 2,810 m a.s.l.): 317.
Peru: 19) Baños del Inca, Cajamarca (07°12′54″ N; 78°26′08″ W; 2,611 m a.s.l.): 72; 20) Rio Lurin, Lima (12°12′28″ S; 76°52′00″ W; 154 m a.s.l.): 23.

Lymnaeid materials of the species *G. truncatula* from Albufera of Valencia, Spain, and Qued Tiout, Essaouira, Marrakesh, Morocco were used for comparative analyses.

### Snail laboratory cultures

Lymnaeids were transported under isothermal conditions to the laboratory of Valencia. The possible natural infection by fasciolids was always individually verified prior to the launch of laboratory cultures. This was performed by keeping each lymnaeid specimen isolated in a Petri dish containing a small amount of natural water. After 24 h, the presence or absence of motionless metacercarial cysts or moving cercariae was verified in each Petri dish. A few non-infected specimens from each population were used for species classification of each population by means of DNA sequencing processes.

Afterwards, non-infected lymnaeids were arranged in standard breeding boxers containing 2000 ml fresh water according to lymnaeid species classification, to assure pure specific cultures. Finally, snails were adapted to and maintained under experimentally controlled conditions of 20° C, 90% relative humidity and a 12 h/12 h light/darkness photoperiod in precision climatic chambers (Heraeus-Vötsch VB-0714 and HPS-500). The water was changed weekly and lettuce added *ad libitum*.

### Voucher specimens and taxonomic nomenclature aspects

Voucher specimens of *L. schirazensis*, both experimentally raised and molecularly classified as well as from molecularly classified populations collected in the field, have been deposited in the Department of Parasitology, Faculty of Pharmacy, University of Valencia, Spain, and the Staatliche Naturhistorische Sammlungen Dresden, Museum für Tierkunde, Dresden, Germany. Other field-collected specimens are kept at Laboratorio de Biología de Parásitos y Vectores, Escuela de Biología, Benemérita Universidad Autónoma de Puebla, Puebla, México.

The list of species (and subspecies) of molluscs and trematode parasites included in this study, according to the zoological nomenclature, is included in Table S1. The generic name *Lymnaea* is used here throughout in the broad taxonomic meaning (*sensu lato* = *s. l.*).

### Molecular techniques

#### DNA extraction

DNA was extracted from more than one specimen of a given population when this was deemed necessary for sequence verification. Only snails that appeared free of helminth infection were used in the molecular analyses. To reduce the risk of contamination of DNA from helminths further (which are more likely to be localized in other tissues), DNA was only isolated from the foot of each snail. Use of just the feet, rather than all the soft tissues, also prevented the development in the DNA pellets of the white flocculate substance (probably of polysaccharides) and melanic pigments that can inhibit Polymerase Chain Reaction (PCR) and cause amplification of non-specific products [15], [34].

Snail feet fixed in 70% ethanol were used for DNA extraction procedures. After dissection under a microscope, half of the foot was suspended in 400 µl of lysis buffer (10 mM Tris-HCl, pH 8.0, 100 mM EDTA, 100 mM NaCl, 1% sodium dodecyl sulfate SDS) containing 500 µg/ml Proteinase K (Promega, Madison, WI, USA) and digested for 2 hr at 55° C with alternate shaking each 15 min. Steps of the procedure were performed according to methods outlined previously [1], [15], [33]. Total DNA was isolated according to the phenol-chloroform extraction and ethanol precipitation method [47]. Each pellet was dried and resuspended in 30 µl sterile TE buffer (pH 8.0). This suspension was stored at −20° C until use.

#### DNA sequence amplification

DNA sequences were amplified by PCR using 4–6 µl of genomic DNA for each 50 µl PCR reaction, according to methods outlined previously [1], [15], [33], [35]. Each one of the five DNA markers were PCR amplified independently for each lymnaeid specimen and each PCR product was sequenced for a bona-fide haplotype characterization. A set of 8 conserved oligonucleotide primers was used for the amplification of five superimposed fragments of the 18S ribosomal RNA gene using specific primers and a standard protocol [15], [34] to amplify specific 18S rDNA regions. Ribosomal DNA spacers ITS-2 and ITS-1 were amplified using primers designed in conserved positions of 5.8S and 28S rRNA genes and 18S and 5.8S rRNA genes of several eukaryote Metazoa species, respectively [1], [15], [39]. The target 16S gene region was amplified by PCR using a set of universal primers [48]. Amplification procedures and thermal cycler conditions were carried out as previously described for lymnaeids [42]. A *cox*1 gene fragment was amplified using universal primers [49]. Amplifications were generated in a Mastercycle ep*gradient* (Eppendorf, Hamburg, Germany), by 30 cycles of 30 sec at 94° C, 30 sec at 50° C and 1 min at 72° C, preceded by 30 sec at 94° C and followed by 7 min at 72° C for ITS-2 and ITS-1, and by 40 cycles of 30 sec at 90° C, 1 min at 48° C and 1 min at 72° C, preceded by 2.5 min at 94° C and followed by 10 min at 72° C for *cox*1. Ten µl of each PCR product was checked by staining with ethidium bromide on 1% Nusieve® GTG agarose (FMC) gel electrophoresis, using Molecular Weight Marker VI (Boehringer Mannheim) at 0.1 µg DNA/µl as control.

#### Purification and quantification of PCR products

Primers and nucleotides were removed from PCR products by purification with Wizard™ PCR Preps DNA Purification System (Promega, Madison, WI, USA) according to the manufacturer′s protocol and resuspended in 50 µl of 10 mM TE buffer (pH 7.6). The final DNA concentration was determined by measuring the absorbance at 260 and 280 nm.

#### DNA sequencing

The sequencing of the entire 18S rRNA gene, the complete rDNA ITS-2 and ITS-1, and the fragments of the mtDNA 16S and *cox*1 genes was performed on both strands by the dideoxy chain-termination method [50]. It was carried out with the Taq dye-terminator chemistry kit for ABI 373A and ABI 3700 capillary system (Perkin Elmer, Foster City, CA, USA), using PCR primers.

### DNA haplotype nomenclature

The codes for the sequences obtained follow the standard nomenclature previously proposed for lymnaeid snails [12], [16], [39]. Note that haplotype codes are only definitive in the case of complete sequences. When dealing with fragments or incomplete sequences, haplotype codes are provisional.

### Software programs

#### Sequence alignments

Sequences were aligned using CLUSTAL-W version 1.8 [51] and MEGA 4.0 [52], and assembly was made employing Staden Package [53]. Subsequently, minor corrections were manually introduced for a better fit of nucleotide correspondences in microsatellite sequence regions. Homologies were performed using the BLASTN programme from the National Center for Biotechnology information website (http://www.ncbi.nlm.nih.gov/BLAST). Genetic distances were measured using parameters provided by PAUP v.4.0 b10 [54].

#### Sequence comparisons

The following sequences from GenBank-EMBL were used for comparative analyses (in the following, names of taxa according to articles in which sequences were described; see Table S1 for systematic-taxonomic notes):

18S rRNA gene: complete sequences of *Lymnaea* (*Lymnaea*) *stagnalis* (GenBank Accession Number Z73984), *Lymnaea* (*Stagnicola*) *palustris* (Z73983), *Omphiscola glabra* (Z73982), and *Galba truncatula* (Z73985) [33]; *L. cubensis* (Z83831) [15], [34], *L. viatrix* and *L. neotropica* (both species with the same sequence AM412222) [15], and *L. humilis* (FN182190) [45], all four from respective type localities; *L. cousini* and *L. meridensis* (both species with the same sequence FN598151), and *Pseudosuccinea columella* (FN598152) [55]; *Radix auricularia (*Z73980) and *R. balthica* (Z73981) [33]. Other incomplete sequences available at GenBank were not used to avoid problems in comparative sequence analyses.
rDNA ITS-2: *L.* (*S.*) *palustris palustris* (AJ319620), *L*. (*S*.) *palustris turricula* (AJ319618), *L*. (*S*.) *fuscus* (AJ319621), and *Catascopia occulta* (AJ319642) [1], [14]; *C. catascopium* (AF013143), *C. emarginata (*AF013141, AF013142), *C. elodes* (AF013138), and *Hinkleyia caperata* (AF013139) [38]; *G. truncatula* H1 (AJ296271), H2 (AJ243017), and H3 ( = *L. viatrix sensu* Ueno et al., 1975; = *L. cubensis sensu* Ueno et al., 1975) (AJ272051) [1], [15], [35]; *L. cubensis* (AM412223), *L. viatrix* (AM412224), *L. neotropica* (AM412225) [15], *L. humilis* (FN182191) [45], *L. cousini* (FN598153), and *L. meridensis* (FN598154) [55], all six sequences from respective type localities; *P. columella* (FN598156) [55].
rDNA ITS-1: *L.* (*S.*) *palustris palustris* (AJ626849), *L*. (*S*.) *palustris turricula* (AJ626853), *L*. (*S*.) *fuscus* (AJ626856), and *C. occulta* (AJ626858) [39]; *C. catascopium* (AF013143), *C. emarginata* (AF013142), *C. elodes* (AF013138), and *Hinkleyia caperata* (AF013139) [38]; *G. truncatula* HA (AJ243018), HB (AJ296270), and HC ( = *L. viatrix sensu* Ueno *et al*., 1975; = *L. cubensis sensu* Ueno et al., 1975) (AJ272052) [1], [15], [35], [39]; *L. cubensis* (AM412226), *L. viatrix* (AM412227), *L. neotropica* (AM412228) [15], *L. humilis* (FN182193) [45], *L. cousini* (FN598157), and *L. meridensis* (FN598159) [55], all six sequences from respective type localities; *P. columella* (FN598160) [55].
mtDNA 16S rRNA gene: *Fossaria bulimoides* (AF485657), *F. obrussa* (AF485658), *S. elodes* (AF485652), and *Stagnicola bonnevillensis* (AF485655) [43]; *F. bulimoides* (EU038315) and *S. elodes* isolate 44106 (EU038305) [56]; *L. cubensis* (FN182204) and *L. humilis* (FN182195) [45].
mtDNA *cox*1 gene: *S. elodes* (EU038352) [56]; *S. elodes* (AY227368), *F. bulimoides* (AY227367), and *Austropeplea tomentosa* (AY227365) [44]; *G. truncatula* from Spain (AM494011) [15] and Germany (EU818799) [57]; *L. cubensis* from Cuba (AM494009–type locality) [15] and the USA (FN182205) [45], *L. viatrix* (AM494010–type locality), *L. neotropica* from Peru (AM494008–type locality) [15] and Argentina [58], *L. humilis* (FN182197-9–type locality) [45], *L. cousini* (FN598161–type locality, and FN598162-3) and *L. meridensis* (FN598164–type locality) [55]; *P. columella* from Australia (AY227366) [44] and Puerto Rico (FN598165) [55].

#### Representation of the 18S rRNA secondary structure

The previously published secondary structure prediction for *Limicolaria kambeul* 18S rRNA [59], based on the general eukaryote 18S rRNA secondary structure [60], was used and extended to encompass lymnaeid sequences.

### Phylogenetic inference

Phylogenies were inferred from DNA sequences using maximum-likelihood (ML) estimates with PAUP [54]. ML parameters such as model, base frequencies, transition/transversion ratio (ts/tv), the shape parameter for the gamma distribution, and the proportion of invariant sites, were optimized using the hierarchical likelihood ratio test (hLRT) and the Akaike information criterion (AIC) [61,62), implemented in Modeltest 3.7 [63]. Starting branch lengths were obtained using the least-squares method with ML distances.

To provide an assessment of the reliability of the nodes in the ML tree, three methods were used. First, a bootstrap analysis using 1000 replicates was made with heuristic search in PAUP. Second, a distance-based phylogeny using the neighbour-joining (NJ) algorithm [64] with ML pairwise distances was obtained. Statistical support for the nodes was evaluated with 1,000 bootstrap replicates, with and without removal of gapped positions. Third, a Bayesian phylogeny reconstruction procedure was applied to obtain posterior probabilities (BPP) for the nodes in the ML tree, by using the same evolutionary model as above, implemented in MrBayes 3.1 [65] with four chains for 1,000,000 generations and trees were sampled every 100 generations. The first 1,000 trees sampled were ruled out (“burn-in”), and clade posterior probabilities (PP) were computed from the remaining trees. Alternative methods of phylogenetic reconstruction allowing for an evaluation of the support for each node were also applied. A distance-based phylogeny using the NJ algorithm with LogDet distances was obtained. Statistical support for the nodes was evaluated with 1,000 bootstrap replicates.

Due to different limitations recently shown by mtDNA markers for interspecific sequence analyses in invertebrates [46], [66], [67], phylogenetic reconstruction by combined sequences data sets was made from ribosomal and mitochondrial markers separately. Combined sets analysed were: a) ITS-1 and ITS-2, considered as the markers to fit best for the analysis of relationships between species, as has already been verified in Lymnaeidae [16]; b) 18S, ITS-1 and ITS-2, to increase the support for evolutionarily older divergence nodes; c) 16S and *cox*1, to evaluate mtDNA genome information; and d) *cox*1 gene, by using both the three and only the first two codon positions to assess potential saturation.

Phylogenetic analyses were performed using reference sequences of lymnaeid DNA (see species and Acc. Nos. in list noted above in chapter of sequence comparisons), after adding the following sequences stored at databases: ITS-2: *Radix auricularia* H1 (AJ319628) and *R. balthica* H1 (AJ319633); ITS-1: *R. auricularia* HA (JF922878) and *R. balthica* HA (JF922879); 16S: *L.* (*L.*) *stagnalis* (AF485659), *L.* (*S.*) *palustris* (U82082), *G. truncatula* (HQ283236), *L. viatrix* (HQ283239), *L. humilis* HA and HB (FN182195, FN182196), *L. cousini* (HQ283237), *L. meridensis* HA, *P. columella* (U82073) and *R. auricularia* (AF485659). *cox*1: *L.* (*L.*) *stagnalis* (EU818795), *L.* (*S.*) *palustris* (EU818801), *R. auricularia* (EU818800). The intergenic region sequence (AY030361) [68] including both ITSs of a planorbid species, *Biomphalaria pfeifferi*, was used as outgroup. For the combined sets using 18S and 16S, *B. alexandrina* 18S (BAU65225), ITSs (AY030371) and 16S (AY030204) [68] were used for outgroups, similarly as *B. alexandrina cox*1 (AF199110).

### Phenotypic study

Only specimens from pure laboratory cultures experimentally maintained in climatic chambers and with previous molecularly assessed lymnaeid species classification were used for snail description and intraspecific variability studies.

Shells and egg clusters of lymnaeids were measured, according to traditional malacological methods [19], using a computerised image-analysis system (CIAS) [69]. This system is based on a DXC-930P colour video camera (Sony, Tokyo) fitted to a stereomicroscope, and connected to computer running image analysis software (ImageProH Plus 4.5; Media Cybernetics Inc., Silver Spring, MD).

For anatomical studies, adult lymnaeids were collected in the field and allowed to relax overnight in water containing menthol. They were then immersed in hot water (70° C) for 40 s before transfer to water at room temperature. The soft parts were drawn from the shells with forceps applied to the cephalopedal mass, and fixed in slightly modified Railliet–Henry's fluid (930 ml distilled water, 6 g NaCl, 50 ml 40% formalin, and 20 ml glacial acetic acid). The fixed snails were then dissected under a stereomicroscope, so that drawings of the reproductive system could be made using a camera lucida.

Egg clusters were obtained from living lymnaeids experimentally maintained in climatic chambers. Living egg clusters were measured with CIAS. For egg clusters, cluster roundness (CR = CP^2^/4πCA) measurements were used to quantify the cluster shape. It is a measure of how circular an object is (the expected perimeter of a circular object divided by the actual perimeter). A circular object will have a roundness of 1.0, while more irregular objects will have larger values [70].

### Experiments for selfing verification and characterisation

To verify the capacity of snails to give rise to offspring by selfing ( = autofecundation), egg clusters laid inside the breeding containers, in which pure laboratory cultures were kept within the climatic chambers, were isolated in Petri dishes. Immediately after egg hatching, each newborn snail specimen was isolated in a small Petri dish provided with water and microalgae (*Oscilatoria formosa*) as food. The growth of the snail and its possible egg cluster laying were thereafter followed on a daily basis. Small portions of fresh lettuce were added to complement the food diet when lymnaeid size was sufficient. This follow-up was continued not only until the first egg cluster appeared in the Petri dish, but expanded for up to several weeks until snail death. The purpose was to obtain numerous egg clusters from each isolated snail and to analyse their laying capacity. The following characteristics were assessed: snail life span ( = days elapsed from day of hatching to day of death); prelaying period ( = days elapsed from day of hatching to day when first cluster was laid); laying period ( = days of sexual activity elapsed from first to last day when clusters were laid by a snail specimen, inclusive); postlaying period ( = days elapsed between last laying day and death); total laying capacity ( = number of clusters/life span in days); laying rate in the sexually active period ( = number of clusters/laying period in days). Shape and size of the living clusters were measured with CIAS, and egg numbers per cluster were noted to verify a potential correlation with the size and age of the respective snail individual at the day of laying.

### Liver fluke experimental infection assays

Isolates of *F. hepatica* from Poland and Peru, and *F. gigantica* from Egypt and Vietnam, maintained in the laboratory according to previously described methods [35], were used for experimental infection assays of the laboratory-reared lymnaeids.

Liver fluke eggs in fresh water were maintained in complete darkness at 20° C to start the embryogenic process. Embryogenesis was followed at intervals of four days until fully embryonated containing a developed miracidium. Developed miracidia were forced to hatch by putting fully embryonated eggs under light and used for the experimental infection of snails. Snails of the geographical strains from El Kazza and Tiba in Egypt and from Albufera of Valencia in Spain were used for experiments. These lymnaeid strains were selected due to their higher adult survival rates under experimental conditions. Only laboratory-borne specimens were used. Snails of different size and age within the length range of 3.5–7.5 mm were used to assess infection susceptibility.

Lymnaeids were mono- or trimiracidially infected by the aforementioned *F. hepatica* and *F. gigantica* isolates, by exposing each snail to one or three miracidia for four hours in a small Petri dish containing 2 ml of fresh water. During this short period, snail specimens were forced to stay inside water and the disappearance of the miracidium was taken as verification of its successful penetration into the snail. Therefafter, snails were returned to the same standard conditions in the climatic chamber (2000 ml containers, 20° C, 90% r.h., 12 h/12 h light/darkness, dry lettuce *ad libitum*) until day 30 post-infection (dpi). In that day, they were once again isolated in Petri dishes to permit daily monitoring of potential cercarial shedding by individual snails. Lettuce was provided *ad libitum* to each snail in a Petri dish during this monitoring period until death of the snail.

## Results

### DNA sequences

Sequence characteristics are noted in Table 1. Data includes length and GC/AT content, obtained for each one of the five DNA markers analysed in the populations of *L. schirazensis* from the twenty localities of eight countries (Figure 1) with their corresponding haplotype codes and GenBank Accession Nos.

**Table 1:** Nuclear ribosomal DNA and mitochondrial DNA marker sequences obtained from populations of *Lymnaea schirazensis* and respective GenBank accession numbers.

DNA marker	Populations	Haplotype No.	Sequence length (nucleotide No.)	GC or AT content (%)	Accession No.
rDNA 18S	all populations studied	L.schir-18S-H1	1852 bp	GC 51.4	FR772291
rDNA ITS-2	Iran, Egypt, Spain, the Dominican Republic, Venezuela, and one population from Peru (Baños del Inca)	L.schir-H1	436 bp	GC 53.9	JF272601
	Mexico, Ecuador and one population from Peru (Rio Lurin)	L.schir-H2	444 bp	GC 53.8	JF272602
rDNA ITS-1	Iran, Egypt, Spain, the Dominican Republic and Venezuela	L. schir -HA	531 bp	GC 56.1	JF272603
	Mexico, Ecuador and Peru	L. schir -HB	533 bp	GC 55.9	JF272604
mtDNA 16S rRNA	all populations except one	L.schir-16S-HA	421 bp	AT 69.6	JF272605
	one population from the Dominican Republic (Constanza)	L.schir-16S-HB	421 bp	AT 69.6	JF272606
mtDNA *cox*1	Iran, Egypt, Spain, the Dominican Republic, Mexico (populations from Los Molinos, Escuela A. Obrego in La Trinidad Tepango, and Jiutepec), Venezuela, Ecuador (population from Machachi) and Peru (population from Rio Lurin)	L.schir-*cox*1-Ha	672 bp	AT 69.5	JF272607
	one population from Mexico (Xalpatlaco in Atlixco, Puebla)	L.schir-*cox*1-Hb	672 bp	AT 69.3	JF272608
	one population from Ecuador (La Buena Esperanza)	L.schir-*cox*1-Hc	672 bp	AT 69.6	JF272609
	one population from Peru (Baños del Inca)	L.schir-*cox*1-Hd	672 bp	AT 69.1	JF272610

Nuclear rDNA genes and spacers concern complete sequences and definitive haplotype numbers; mtDNA genes only sequence fragments and provisional haplotype numbers. H = haplotype.

### 18S rRNA gene

**Figure 2:**
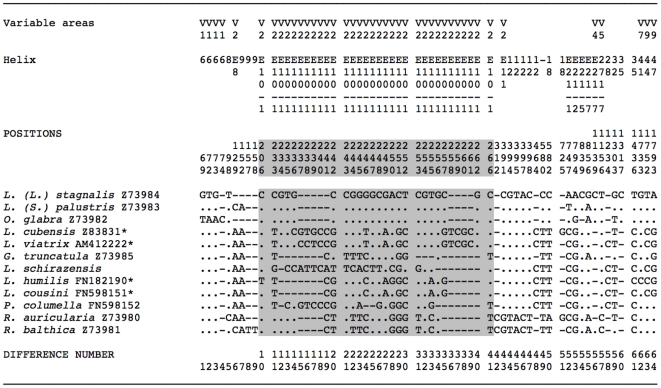
Nucleotide differences in a total of 64 variable positions found in the complete 18S rDNA sequence of the lymnaeid species compared and their location in the secondary structure. Helix, Position and Difference number = numbers to be read in vertical. Position = numbers refer to positions obtained in the alignment made with MEGA 4.0. Identical = .; Indel = −. Shaded area corresponds to Helix E10-1 of the variable area V2 where differences in the 18S rRNA gene of Lymnaeidae are concentrated [33]. GenBank Accession Nos. = Z73980−Z73985 [33]; Z83831 [34]; AM4122222 [15]; FN182190 [45]; FN598151- FN598152 [55]; *L. schirazensis* from present paper. Sequence correspondences: * *L. cubensis*, *L. viatrix*, *L. humilis* and *L. cousini* without definitive genus ascription; 18S identical in *L. viatrix* and *L. neotropica* [15]; 18S identical in *L. cousini* and *L. meridensis* [55].

All of the 20 populations of *L. schirazensis* presented the same 18S sequence (Table 1). When comparing this 18S sequence with that of *G. trucatula*, a total of 19 variable positions appeared. They included 6 ts, 2 tv and 11 insertions/deletions (indels) in a 1853 bp-long pairwise alingment, most differences being located in the variable Helix E-10 of the V2 region according to the secondary structure (Figure 2).

A multiple 18S sequence alignment was 1867 bp long. It included: several *Galba/Fossaria* vector species such as *L. cubensis*, *L. viatrix* (the sequence being identical to that of *L. neotropica*), *G. truncatula, L. humilis*, and *L. cousini* (sequence identical to that of *L. meridensis*); the peculiar species *P. columella*; three representatives of stagnicolines (*L.* (*L.*) *stagnalis*, *L.* (*S.*) *palustris* and *O. glabra*); and two of the *Radix* group (*R. auricularia* and *R. balthica*). This alignment showed a total of 64 variable nucleotide positions (3.43% nucleotide divergence). Thirty-two of these 64 polymorphic sites appeared grouped in the short sequence between positions 206 and 266, which corresponds to the helix E10-1 of the variable area V2 of the secondary structure (Figure 2).

### Second internal transcribed spacer rDNA ITS-2

Two different ITS-2 haplotypes were found among the *L. schirazensis* populations analysed (Table 1). Their pairwise comparison showed only 8 polymorphic sites, corresponding to 8 indels caused by the tetranucleotide microsatellite repeat TGCT, present twice in haplotype 1 between positions 128 and 135 of the alignment but absent in haplotype 2.

The very high number of nucleotide differences detected in the pairwise comparisons of these two ITS-2 sequences with the three ITS-2 haplotypes of *G. truncatula* available at GenBank is noteworthy. In the different alignments performed, with a bp length varying between 469 to 477, a total of 130–139 differences appeared, including 18–19 ts, 11 tv and 101–109 indels (Table 2).

**Table 2:** Sequence differences detected in pairwise comparisons of ITS-2 and ITS-1 between *Lymnaea schirazensis* and the most morphologically similar species *Galba truncatula*.

	Alignment length	Total differences		Substitutions				Insertions+deletions	
				Transitions		Transversions			
Compared species	No. of bp	No.	%	No.	%	No.	%	No.	%
ITS-2:									
*L. schirazensis* H1 vs. *G. truncatula* H1	477	139	29.14	19	3.98	11	2.31	109	22.83
*L. schirazensis* H1 vs. *G. truncatula* H2	477	138	28.93	18	3.77	11	2.31	109	22.83
*L. schirazensis* H1 vs. *G. truncatula* H3	477	139	29.14	19	3.98	11	2.31	109	22.83
*L. schirazensis* H2 vs. *G. truncatula* H1	469	131	27.93	19	4.05	11	2.34	101	21.53
*L. schirazensis* H2 vs. *G. truncatula* H2	469	130	27.72	18	3.84	11	2.34	101	21.53
*L. schirazensis* H2 vs. *G. truncatula* H3	469	131	27.93	19	4.05	11	2.34	101	21.53
ITS-1:									
*L. schirazensis* HA vs. *G. truncatula* HA	560	136	24.28	31	5.53	22	3.93	83	14.82
*L. schirazensis* HA vs. *G. truncatula* HB	560	137	24.46	32	5.71	22	3.93	83	14.82
*L. schirazensis* HA vs. *G. truncatula* HC	560	138	24.64	32	5.71	23	4.11	83	14.82
*L. schirazensis* HB vs. *G. truncatula* HA	558	134	24.01	32	5.73	21	3.76	81	14.52
*L. schirazensis* HB vs. *G. truncatula* HB	558	135	24.19	33	5.91	21	3.76	81	14.52
*L. schirazensis* HB vs. *G. truncatula* HC	558	136	24.37	33	5.91	22	3.94	81	14.52

When comparing the *L. schirazensis* ITS-2 sequences with the other species of the *Galba*/*Fossaria* group, the pairwise ITS-2 distance matrix obtained with PAUP (only parsimony informative sites considered) showed that the numbers of nucleotide differences were unexpectedly high in all cases. The lowest of these numbers appeared when comparing with *G. truncatula*, although rather high (64–66). Regarding *L. humilis*, *L. cousini* and *L. meridensis*, it ranged from 99 to 118. It appeared to be similarly high (121–136) with regard to *L. cubensis*, *L. viatrix* and *L. neotropica*. Nucleotide differences were also numerous when *L. schirazensis* was compared with stagnicolines and *Pseudosuccinea* (Table S2).

### First internal transcribed spacer rDNA ITS-1

Two different ITS-1 haplotypes were also found among the *L. schirazensis* populations analysed (Table 1). The pairwise comparison of these two sequences showed only 3 polymorphic sites, corresponding to two insertions in positions 296 and 297 (AA or --) and to one mutation in position 443 (A or T) in haplotypes A and B, respectively.

A very high number of nucleotide differences also appeared in the pairwise comparisons with the three ITS-1 haplotypes of *G. truncatula*. In the different alignments performed, with a bp length varying from 558 to 560, a total of 134–138 differences appeared, including 31–32 ts, 21–23 tv and 81–83 indels (Table 2).

When comparing *L. schirazensis* ITS-1 sequences with the other species of the *Galba*/*Fossaria* group, the pairwise ITS-1 distance matrix obtained with PAUP showed that the numbers of nucleotide differences were also very large. The comparison with *G. truncatula* furnished the second lowest number (81–84), after that of the comparison with *L. meridensis* (77–80). Regarding *L. humilis* and *L. cousini*, it ranged from 87 to 92, and it appeared to be similarly high (83–92) with regard to *L. cubensis*, *L. viatrix* and *L. neotropica*. The number of nucleotide differences increased considerably when *L. schirazensis* was compared with stagnicolines (132–143) and *Pseudosuccinea* (129–130) (Table S3).

### 16S rRNA gene of the mtDNA

Two different sequences of the 16S fragment were found (Table 1). They differred by only 2 mutations (C/A in position 85 and A/G in position 305 of their pairwise alignment, in HA/HB, respectively).

**Figure 3:**
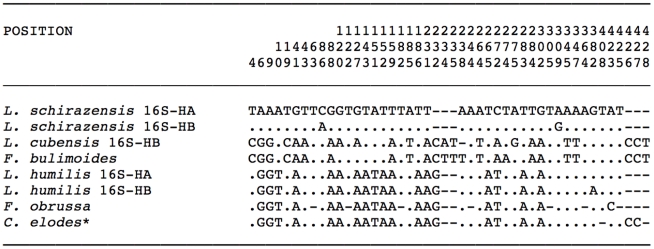
Variable positions showed by the mtDNA 16S sequence fragment in a 433-bp-long alignment including the two haplotypes of *Lymnaea schirazensis* and other *Galba/Fossaria* species. Numbers (to be read in vertical) refer to positions obtained in the alignment made with MEGA 4.0. Identical = . ; Indel = − . Haplotype codes only provisional due to incomplete sequences of the gene. *L. cubensis* 16S-HB (FN182204), *L. humilis* 16S-HA (FN182195) and *L. humilis* 16S-HB (FN182196) [45]; *F. bulimoides* (AF485657) and *F. obrussa* (AF485658) [43]; * the sequence ascribed to the stagnicoline *C. elodes* (EU038305) [56] concerns in fact a *Galba*/*Fossaria* species-see analysis in [45].

Nucleotide comparison with other *Galba/Fossaria* species showed a total of 46 variable positions (10.75%), of which 31 were mutations (7.24%) and 15 were indels (3.50%). This alignment demonstrated that *L. schirazensis* was different from any other *Galba/Fossaria* at the level of this mtDNA gene (Figure 3). In pairwise comparisons, minimum differences were 18 mutations (4.2%) when compared with *L. humilis*, and a maximum of 22 mutations (5.14%) appeared in comparisn with *L. cubensis*. Nucleotide differences were more numerous when comparing with the stagnicolines *S. elodes* and *S. bonnevillensis*. The four species alignment showed a total of 56 variable positions (12.93%), 47 of which were mutations (10.85%) and 9 indels (2.08%) (alignment not shown).

### mtDNA cytochrome c oxidase subunit I *cox*1

Four different *cox*1 sequences were obtained (Table 1). When aligned, a total of 661 positions were conserved and only 11 were variable, comprising 2 parsimony informative and 9 singleton sites (Table 3). When comparing with species of the *Galba*/*Fossaria* group and other proximal lymnaeid species available at GenBank, whose *cox*1 fragment sequences were similar in length to those obtained in the present study, the higher number of nucleotide differences appeared evident in a pairwise *cox*1 distance matrix (Table S4). A comparison with more distant species such as stagnicolines (*S. elodes*) and *Radix* group (*Austropeplea tomentosa*) was not necessary.

**Table 3:** Nucleotide differences found in the sequences of the 672-bp-long mtDNA *cox*1 gene fragment of the four *L. schirazensis* haplotypes described.

Positions		2	2	2	3	3	3	4	4	5	6
	6	7	7	8	3	4	6	1	5	2	5
	3	1	3	7	9	5	0	7	9	2	1
*L.schirazensis cox*1-a	C	C	G	T	T	T	A	T	G	G	C
*L.schirazensis cox*1-b	T	.	C	.	C	C	.	.	.	.	.
*L.schirazensis cox*1-c	.	.	C	.	C	C	.	.	A	A	T
*L.schirazensis cox*1−d	.	T	.	C	.	C	G	C	.	.	.

Positions = numbers (to be read in vertical) refer to variable positions obtained in the alignment made with MEGA 4.0; . = identical to nucleotide in first line; haplotype codes only provisional due to incomplete sequences of the gene.

**Figure 4:**
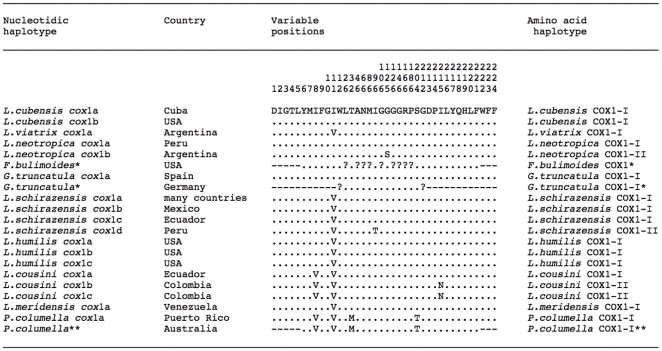
COX1 amino acid sequence differences detected in the alignment of the haplotypes of the lymnaeid species studied, together with species of the *Galba*/*Fossaria* group and other proximal lymnaeid species available in GenBank. Only *cox*1 sequence fragments of a lenght similar to that of sequences obtained in present paper are included. Variable positions = Numbers (to be read in vertical) refer to positions obtained in the alignment made with MEGA 4.0. − = position not sequenced; ? = undetermined amino acid. Haplotype codes only provisional due to incomplete sequences of the gene. * Sequences somewhat shorter and including a few undetermined amino acids; ** sequences somewhat shorter although presumably identical to haplotype *cox*1a of the same species.

The amino-acid sequence of that COX1 gene fragment was 224 aa long. A pairwise comparison showed a 100% identity between the three *L. schirazensis* haplotypes *cox*1-a, *cox*1-b and *cox*1-c, and only one amino-acid difference (isoleucine/threonine, respectively) in position 96 with *L. schirazensis* haplotype *cox*1-d from Baños del Inca, Peru. The provisional haplotypes COX1-I and COX1-II were assigned, respectively. The comparison with the amino acid sequence of other species of the *Galba*/*Fossaria* group and other proximal lymnaeid species showed that COX1-I of *L. schirazensis* was identical to that of other species, such as *L. viatrix*, *L. humilis* and *L. meridensis*. However, *L. schirazensis* COX1-II appeared to be unique (Figure 4).

### Phylogenetic analysis

#### Combined ITS-1 and ITS-2 sets

**Figure 5:**
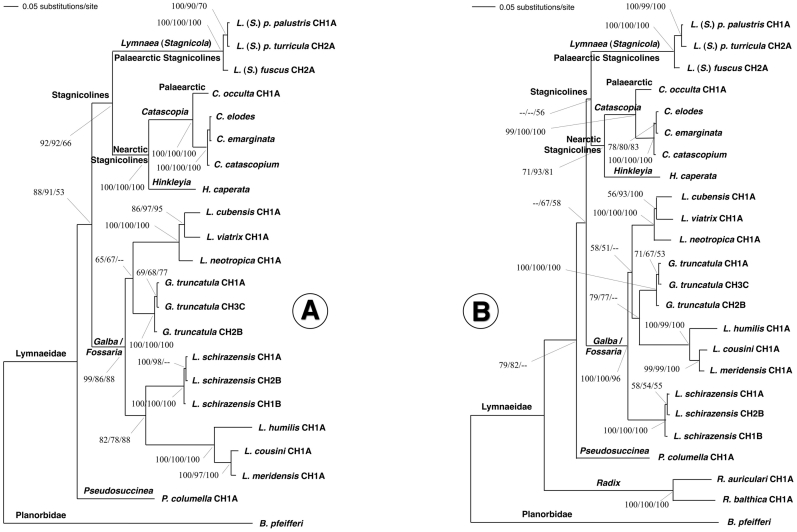
Phylogenetic trees of lymnaeid species studied based on maximum-likelihood (ML) estimates: A) data set of ITS-1 and ITS-2, with the planorbid *B. pfeifferi* as outgroup (−Ln = 10016.27013); B) same data set of ITS-1 and ITS-2 with *B. pfeifferi* as outgroup, after adding *Radix* species (−Ln = 10078.46520). Scale bar indicates the number of substitutions per sequence position. Support for nodes a/b/c: a: bootstrap with NJ reconstruction using PAUP with ML distance and 1000 replicates; b: bootstrap with ML reconstruction using PAUP with 1000 heuristic replicates; c: Bayesian posterior probability with ML model using MrBayes. See Table S1 for systematic-taxonomic notes.

This single data-set generated a robust tree, indicating phylogenetic accordance between the two spacers. The ML model best fitting this data-set was HKY85+I, using a ts/tv ratio of 2 (kappa = 3.8979086), base frequencies for A, C, G and T of 0.21534, 0.26862, 0.22661 and 0.28943, respectively, and a proportion of invariable sites = 0.087. To assess the reliability of the nodes in the ML tree (Figure 5 A), a bootstrap analysis using 1,000 replicates was made using branch-swapping algorithm (tree-bisection-reconnection TBR) with heuristic search and the neighbour-joining (NJ) algorithm with the ML pairwise distances in PAUP. Finally, a Bayesian phylogeny reconstruction procedure was applied to obtain posterior probabilities (BPP) for the nodes in the ML tree with MrBayes 3.1.

In the ML tree obtained (Figure 5 A), *P. columella* appeared basal to two large groupings, namely stagnicolines (including both Palaearctic and Nearctic species), and the *Galba*/*Fossaria* clade which comprised the *F. hepatica* main vector species. *Lymnaea schirazensis* appeared included within the latter. However, contrary to what would be expected, *L. schirazensis* did not cluster together with *G. truncatula*, of Old World origin, or other morphologically similar lymnaeids such as the New World *L. cubensis*, *L. viatrix* and *L. neotropica*. Surprisingly, it appeared linked to the Nearctic *L. humilis* and the two Neotropical *L. cousini* and *L. meridensis*, within a relatively well supported branch (82/78/88 in NJ/ML/BBP).

The topology obtained with the NJ algorithm using LogDet distances (figure not shown) was identical to that shown by the ML tree (Figure 5 A).

When adding two *Radix* species (data matrix of 24 taxa with 1,767 characters), the ML model best fitting was HKY85+G+I, using a ts/tv ratio of 1.45 (kappa = 2.872483), base frequencies for A, C, G and T of 0.22764, 0.25354, 0.23176 and 0.28705, respectively, a shape parameter (alpha) = 0.99, and a proportion of invariable sites = 0.13. In the ML tree obtained (Figure 5 B), *Radix* species clustered independently in a branch basal to all the remaining lymnaeids. Unexpectedly, the location of *L. schirazensis* changed, now becoming basal to the other *Galba/Fossaria* members which clustered together although with very low supports. The topology of the NJ LogDet tree was similar (figure not shown), but with *L. schirazensis* in a subclade with *G. truncatula* showing low bootstrap (only 59).

#### Combined 18S, ITS-1 and ITS-2 set

**Figure 6:**
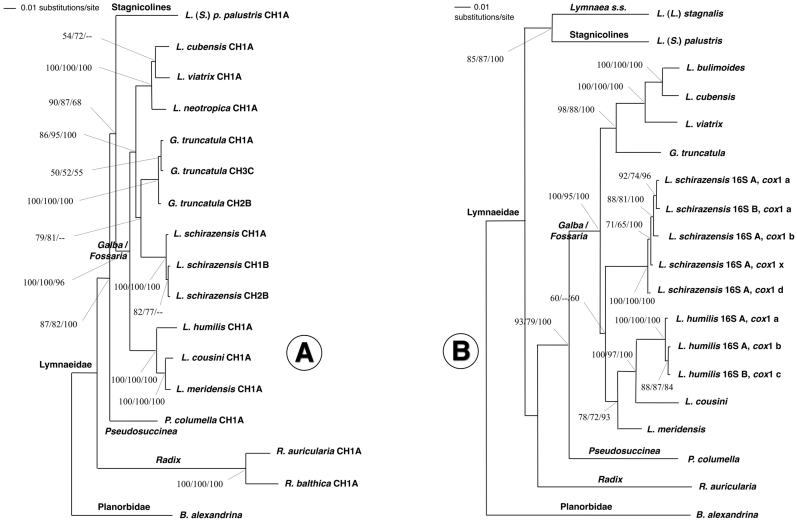
Phylogenetic trees of lymnaeid species studied based on maximum-likelihood (ML) estimates: A) data set of 18S, ITS-1 and ITS-2, with *B. alexandrina* as outgroup (−Ln = 13171.38533); B) data set of 16S and *cox*1, with *B. alexandrina* as outgroup (−Ln = 5282.96177). Scale bar indicates the number of substitutions per sequence position. Support for nodes a/b/c: a: bootstrap with NJ reconstruction using PAUP with ML distance and 1000 replicates; b: bootstrap with ML reconstruction using PAUP with 1000 heuristic replicates; c: Bayesian posterior probability with ML model using MrBayes. See Table S1 for systematic-taxonomic notes.

This three ribosomal marker data set (matrix of 17 taxa and 3,540 characters) also generated a robust tree. The ML model best fitting was HKY85+I, using a ts/tv ratio of 1.48 (kappa = 2.9601982), base frequencies for A, C, G and T of 0.22150, 0.25100, 0.25820 and 0.26930, respectively, and a proportion of invariable sites = 0.44. In the ML tree obtained (Figure 6 A), *L. schirazensis* appeared clustering together with *G. truncatula* (79/81/- in NJ/ML/BPP), and the branch including *L. humilis, L. cousini* and *L. meridensis* appeared as the sister group inside the *Galba/Fossaria* clade. The topology obtained with the NJ algorithm using LogDet distances (figure not shown) was identical.

#### Combined 16S and *cox*1 set

The data matrix analysed contained 19 taxa and 1,118 characters. The ML model best fitting was HKY85+G+I, using a ts/tv ratio of 1.78 (kappa = 5.083228), base frequencies for A, C, G and T of 0.33500, 0.10560, 0.12210 and 0.43730, respectively, a shape parameter (alpha) = 0.39, and a proportion of invariable sites = 0.35. The ML tree (Figure 6 B) showed *L. schirazensis* haplotypes clustering together with the branch of *L. humilis*, *L. cousini* and *L. meridensis*, although with low support values (60/–/60). The topology furnished by the NJ algorithm using LogDet distances (figure not shown) was similar, with that clade supported by a 64% bootstrap.

#### *cox*1 sets

Two phylogenetic reconstructions were performed, one by using complete sequences of the *cox*1 fragment and another after deleting the third codon position. The data matrix contained 28 taxa, with 672 and 448 characters, respectively. In the trees obtained (figures not shown), very low alpha values, paraphylies and low support values in external nodes, as well as inconsistency of relationships between *Radix*, *Pseudosuccinea* and stagnicolines, suggest sequence saturation.Therefore, a saturation effect may also be expected at the level of the relationships of *L. schirazensis* with other *Galba/Fossaria* species, given the very high nucleotide differences at ITS levels. Consequently, care should also be taken when considering phylogenetic results furnished by the aforementioned 16S and *cox*1 single data set.

### Diagnostic description of *Lymnaea* (*s. l.*) *schirazensis* Küster, 1862

#### Type locality

Shiraz, Iran [71].

#### Other localities

See detailed data on localities of Iran, Egypt, Spain, the Dominican Republic, Mexico, Venezuela, Ecuador and Peru in the “Lymnaeid snail material” section above.

#### Shell

**Figure 7:**
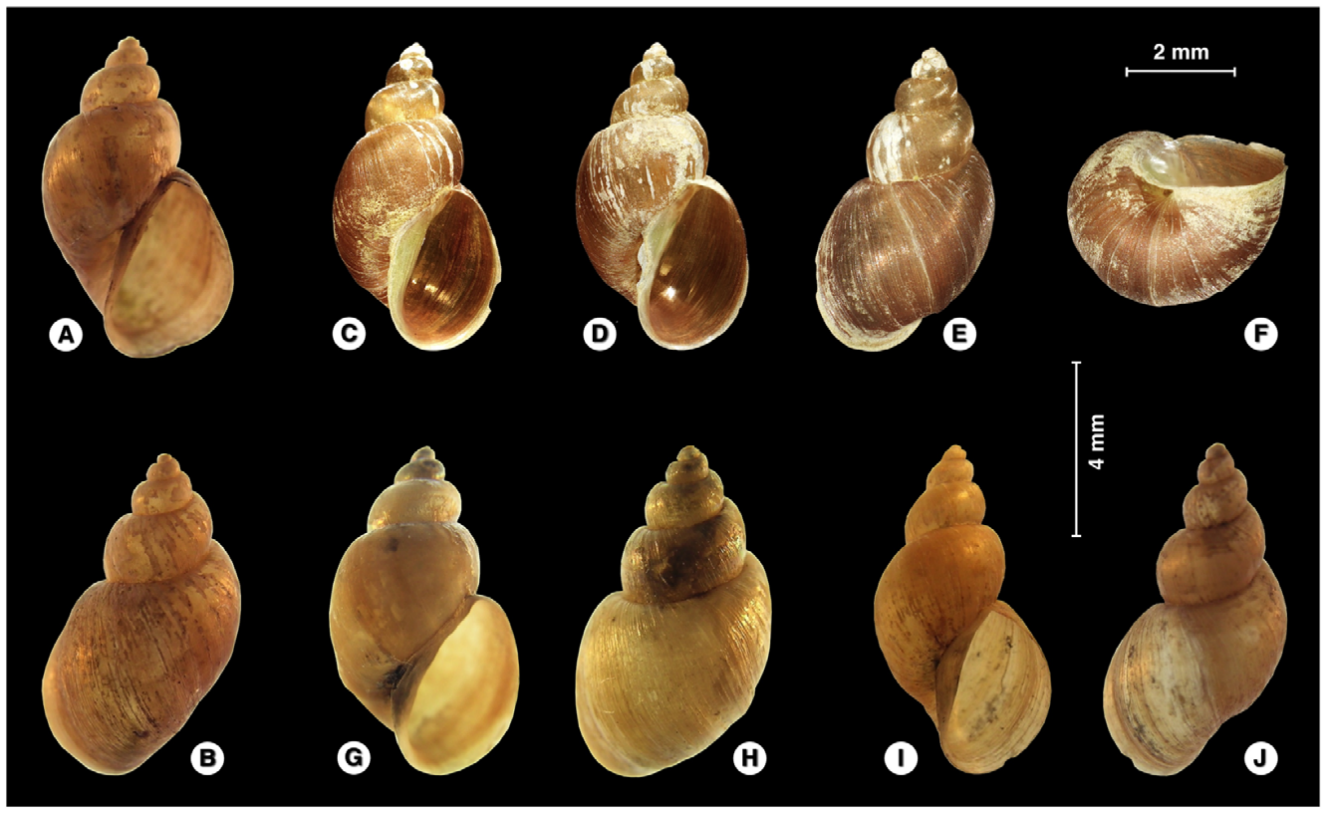
Shells of *Lymnaea schirazensis* in ventral, dorsal and from-below views, showing intraspecific variability: A,B) specimen (7.20 mm high) from Tiba, Delengate district, Behera governorate, Egypt; C) specimen (7.00 mm) from Albufera of Valencia, Valencia province, Spain; D–F) specimen (7.10 mm) from Albufera of Valencia, Valencia province, Spain; G,H) specimen (7.80 mm) from Nules-Moncofar, Castellon province, Spain; I,J) specimen (7.84 mm) from Laguna de Fe y Alegria, El Valle, Estado de Merida, Venezuela. Scale bars: A−E,G−J = 4 mm; F = 2 mm.

The shell was brownish to reddish light brown, thin-walled, elongated conical, usually with four regular convex whorls and up to 5.5 whorls in the longer specimens (Figure 7). The whorls were somewhat inflated, slightly shouldered, with silky and longitudinally striated surface and separated by a deep, well-marked suture, increasing rather slowly in diameter. The columella was straight, unfolded, and the umbilicus open. The last or body whorl was almost ¾ times as high as the shell height, presenting a slight twisting trend along its basal part visible in the biggest shells when viewed dorsally, and which was due to the enlargement of the basal lip of the peristome (in the way of *Pseudosuccinea columella*). The spire was pointed. The aperture was elongatedly oval, slightly oblique, mid-sized and wider at the base. The thin peristome was patent throughout, the umbilicus partially covered by a widened columellar lip. The shell showed a tendency to be approximately one and a half to two times as long as it is wide, and its aperture tends to be slightly less than half as long as the shell. Measurements and calculated ratios of natural and experimental populations were noted (see Table 4 and Table S5, respectively).

**Table 4:** Lymnaeid shell measurement comparison between different natural populations of *Lymnaea schirazensis* and *Galba truncatula* from different countries.

Shell parameters (abbreviation)	Height (SH)	Maximum width (SW)	Aperture length (AL)	Aperture width (AW)	Last spire length (LSL)	Spiral angle (SSA)	Whorl number	SH/SW ratio	SH/AL ratio	SH/LSL ratio
***Lymnaea schirazensis***:										
Iran, Taleb Abad bridge (n = 30)	5.21−7.14 (5.91±0.53)	2.60−3.79 (3.24±0.31)	2.30−3.24 (2.68±0.25)	1.48−2.26 (1.83±0.19)	3.91−5.17 (4.44±0.33)	33.12−61−25 (46.13±6.39)	4−5 (4.27±0.45)	1.59−2.14 (1.83±0.13)	1.95−2.94 (2.24±0.22)	1.25−1.44 (1.33±0.05)
Egypt, El Kazza (n = 30)	5.10−6.91 (5.91±0.45)	2.65−4.11 (3.24±0.32)	2.19−3.12 (2.76±0.23)	1.41−2.29 (1.73±0.20)	4.06−5.40 (4.62±0.34)	27.75−55.20 (43.48±7.22)	4−5 (4.10±0.31)	1.67−2.01 (1.83±0.08)	1.96−2.34 (2.15±0.10)	1.22−1.38 (1.28±0.04)
Egypt, Tiba Delengate (n = 30)	5.45−7.21 (6.40±0.49)	2.87−4.03 (3.50±0.31)	2.33−3.6 (3.02±0.28)	1.45−2.66 (2.03±0.29)	4.21−5.89 (5.03±0.44)	34.36−56.55 (46.89±5.32)	4−5 (4.03±0.18)	1.63−2.13 (1.84±0.10)	1.91−2.42 (2.12±0.12)	1.22−1.37 (1.27±0.03)
Egypt, Bulin El Aly (n = 30)	4.43−6.82 (5.67±0.62)	2.53−3.67 (3.10±0.33)	2.13−3.25 (2.68±0.29)	1.31−1.93 (1.62±0.19)	3.37−5.25 (4.35±0.49)	33.89−56.21 (45.39±5.24)	4−5 (4.13±0.35)	1.62−1.98 (1.80±0.08)	1.95−2.35 (2.08±0.09)	1.22−1.38 (1.28±0.03)
Spain, Albufera Valencia (n = 30)	4.27−7.42 (5.89±0.61)	2.52−4.02 (3.28±0.34)	1.94−3.39 (2.75±0.30)	1.18−2.37 (1.79±0.22)	3.48−5.52 (4.57±0.42)	30.63−57.17 (46.30±5.54)	4−5 (4.17±0.38)	1.59−2.01 (1.80±0.09)	1.90−2.53 (2.15±0.13)	1.21−1.36 (1.29±0.04)
Spain, Castellón (n = 30)	5.25−7.80 (5.99±0.51)	2.92−4.31 (3.32±0.28)	2.56−4.12 (2.85±0.30)	1.48−2.30 (1.73±0.16)	4.12−5.98 (4.59±0.37)	34.61−56.28 (46.58±5.02)	4−5 (4.43±0.50)	1.62−2.18 (1.81±0.11)	1.89−2.31 (2.11±0.09)	1.26−1.35 (1.30±0.02)
Dominican Rep., Constanza (n = 30)	3.50−5.73 (4.94±0.54)	2.09−3.27 (2.80±0.31)	1.74−2.90 (2.50±0.32)	1.20−1.77 (1.52±0.16)	2.92−4.48 (3.87±0.42)	38.74−56.48 (48.09±5.55)	4 (4.00±0.00)	1.50−2.07 (1.76±0.10)	1.76−2.47 (1.98±0.15)	1.18−1.39 (1.27±0.05)
Mexico, Xalpatlaco (n = 33)	2.98−4.25 (3.57±0.38)	1.70−2.47 (2.08±0.18)	1.40−2.29 (1.80±0.23)	0.98−1.47 (1.17±0.12)	2.42−3.51 (2.89±0.30)	26.14−53.54 (41.10±6.15)	3−4 (3.42±0.51)	1.54−1.91 (1.71±0.10)	1.76−2.46 (1.98±0.14)	1.18−1.31 (1.23±0.03)
Mexico, Trinidad Tepango (n = 8)	4.02−5.37 (4.86±0.43)	2.50−3.01 (2.80±0.20)	2.05−2.67 (2.46±0.20)	1.43−1.71 (1.56±0.10)	3.30−4.19 (3.87±0.29)	34.44−45.48 (39.80±3.67)	4−5 (4.13±0.35)	1.61−1.81 (1.74±0.06)	1.93−2.02 (1.98±0.04)	1.22−1.28 (1.26±0.02)
Venezuela, Laguna, El Valle (n = 4)	5.93−8.06 (6.78±0.63)	3.10−4.12 (3.61±0.26)	2.47−3.84 (3.23±0.27)	1.74−2.62 (2.14±0.22)	4.60−6.09 (5.34±0.42)	29.21−55.68 (43.20±5.71)	4−5 (4.20±0.41)	1.66−2.26 (1.88±0.14)	1.78−2.71 (2.11±0.21)	1.19−1.38 (1.27±0.05)
Ecuador, Buena Esperanza (n = 30)	2.41−5.35 (3.37±0.83)	1.39−2.82 (1.77±0.38)	1.16−2.44 (1.62±0.36)	0.77−1.42 (1.00±0.18)	2.06−4.07 (2.68±0.57)	25.05−43.90 (34.52±4.72)	3−4 (3.40±0.50)	1.63−2.05 (1.89±0.10)	1.87−2.38 (2.08±0.15)	1.11−1.33 (1.25±0.05)
Ecuador, Guarandauco (n = 30)	5.76−7.27 (6.30±0.33)	2.88−3.82 (3.25±0.23)	2.53−3.41 (2.91±0.19)	1.46−2.27 (1.71±0.16)	4.45−5.53 (4.85±0.27)	23.66−48.47 (36.34±5.71)	4−5 (4.07±0.25)	1.71−2.15 (1.94±0.09)	1.99−2.41 (2.17±0.12)	1.25−1.35 (1.30±0.03)
Ecuador, Machachi (n = 30)	5.53−6.91 (5.88±0.31)	2.49−3.51 (2.96±0.19)	2.30−3.31 (2.56±0.24)	1.45−1.85 (1.61±0.10)	3.95−5.18 (4.37±0.24)	29.05−43.26 (35.37±3.34)	4−5 (4.23±0.43)	1.78−2.35 (1.99±0.11)	2.05−2.62 (2.30±0.14)	1.30−1.42 (1.35±0.03)
Peru, Baños del Inca (n = 4)	4.00−5.56 (4.79±0.86)	2.04−2.88 (2.47±0.39)	1.82−2.62 (2.20±0.42)	1.19−1.50 (1.35±0.17)	3.07−4.17 (3.62±0.62)	29.13−39.99 (34.57±4.56)	3−4 (3.75±0.50)	1.77−2.03 (1.94±0.12)	2.10−2.22 (2.18±0.05)	1.29−1.34 (1.32±0.02)
Peru, Río Lurín en Lima (n = 2)	3.82−3.89 (3.85±0.05)	1.99−2.20 (2.09±0.15)	1.67−1.78 (1.72±0.08)	1.05−1.14 (1.10±0.06)	3.04−3.08 (3.06±0.02)	32.56−36.52 (34.54±2.80)	4 (4.00±0.00)	1.77−1.92 (1.85±0.11)	2.15−2.34 (2.24±0.13)	1.24−1.28 (1.26±0.02)
***Galba truncatula*** **:**										
Spain, Albufera Valencia (n = 30)	6.79−9.33 (7.73±0.64)	3.70−4.90 (4.29±0.29)	3.30−4.07 (3.58±0.20)	2.05−2.72 (2.40±0.17)	5.28−6.80 (5.78±0.40)	33.94−59.26 (42.73±5.34)	4−5 (4.67±0.48)	1.55−1.96 (1.80±0.09)	1.84−2.38 (2.16±0.14)	1.25−1.47 (1.34±0.05)
Marocco, Oued Tiout (n = 30)	6.57−8.29 (7.58±0.48)	3.79−4.60 (4.26±0.22)	3.29−4.29 (3.86±0.28)	2.40−3.15 (2.77±0.21)	5.19−11.09 (6.05±1.02)	40.91−61.66 50.77±5.37)	4−5 4.30±0.47)	1.64−2.02 (1.78±0.08)	1.84−2.14 (1.97±0.07)	0.69−1.36 (1.27±0.11)

Range include minimum and maximum extremes, with mean±standard deviation SD in parentheses. Measurements in mm. n = number of specimens measured.

#### External morphological characteristics

**Figure 8:**
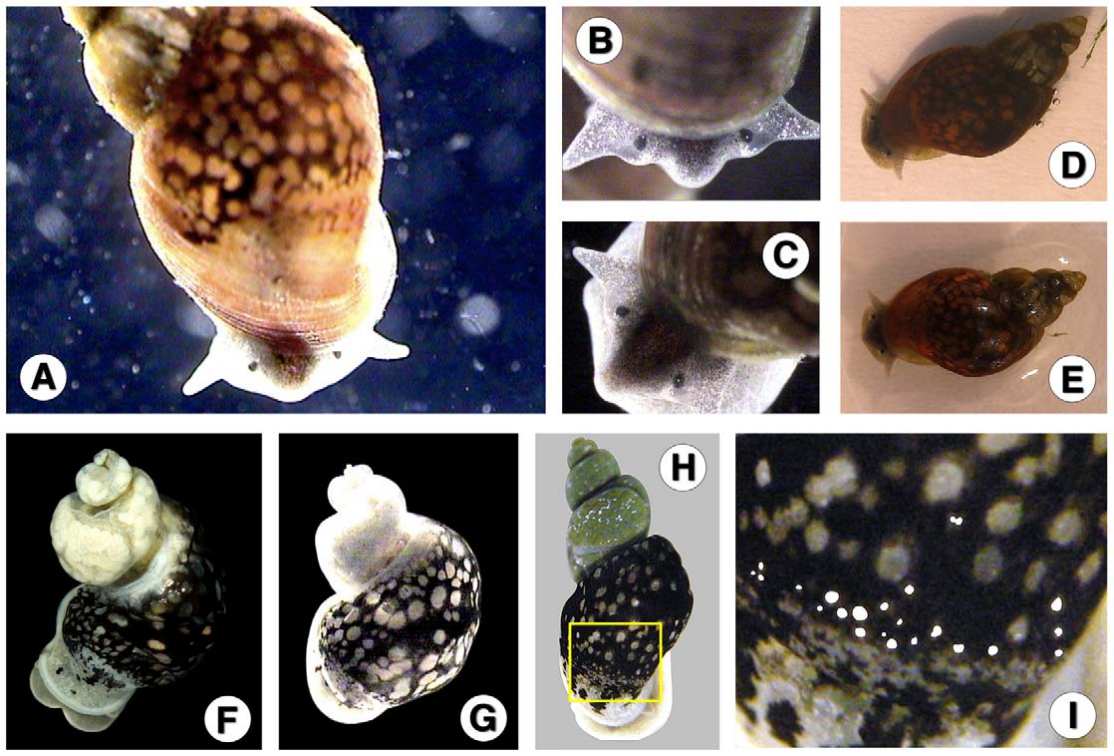
External aspect of *Lymnaea schirazensis*: A–E) living specimens showing (i) large, round, black eyes, (ii) long, slender tentacles and (iii) dark shell (A = lighted from down; B–D = lighted from above; F = epi− and infralighted simultaneously); F–I) dark brown to blackish mantle roof of specimens from Spain (F, G) and Mexico (H, I) showing small unpigmented white-greyish round spots, including several tiny circles (artificially remarked in white with computer effects in I) at the beginning of the border of the pulmonary region (I = yellow rectangle in H).

The cephalopedeal mass was pale greyish. The eyes were black, relatively big in size (Figure 8 A–C). Tentacles were elongate, slender, pyramidal, with narrow base (Figure 8 A–E). The mantle roof was dark, from dark brown to blackish throughout, with small unpigmented white-greyish round spots including several tiny circles at the beginning of the border of the pulmonary region and a few scattered further away in between the initial large round spots (Figure 8 F–I). The border of the mantle was light grey. The black pigmentation of the hypopeplear region of the mantle roof gave a dark appearance to the shell of living specimens by transparency (Figure 8 D,E). This dark appearance did not depend on the characteristics of the natural habitat, as it was maintained across the different laboratory-reared snail generations (quite the opposite of what happens with several darkish populations of other lymnaeid species under experimental conditions).

#### Anatomy

**Figure 9:**
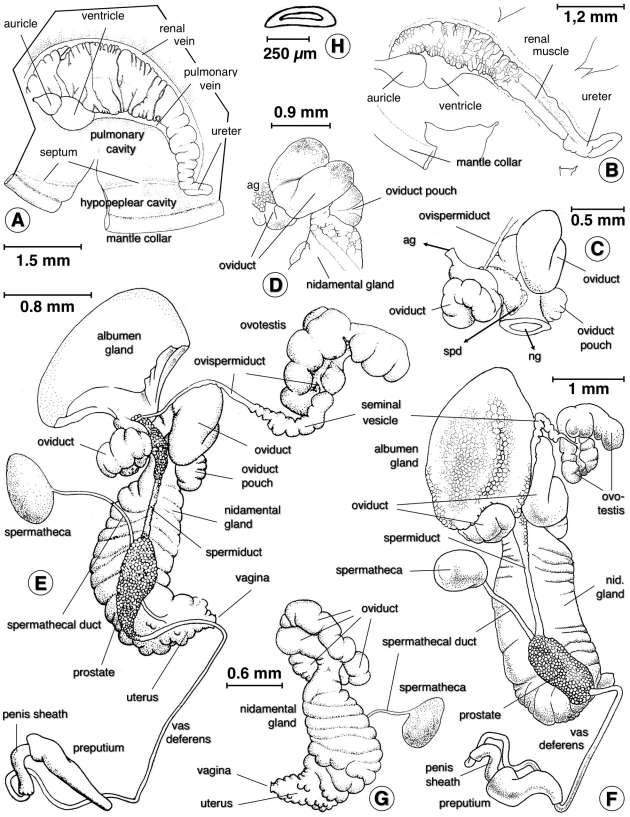
Aspects of soft part anatomy of *Lymnaea schirazensis*: A, B) renal tube and ureter in renal region extending between pericardium and mantle collar; C) carrefour in detail, with arrows indicating ducts to albumen gland (ag), spermiduct (spd) and nidamental gland (ng); D) oviducal crown turned to show detail of the region of oviduct pouch; E, F) reproductive system in two ventral views; G) female complex in dorsal view; H) prostate section showing absence of internal folds. Scale bars: A = 1.5 mm; B = 1.2 mm; C = 0.5 mm; D = 0.9 mm; E = 0.8 mm; F = 1 mm; G = 0.6 mm; H = 250 µm (drawings R. Rojas; plate configuration S. Mas-Coma).

Morphoanatomical features were studied (see Figure 9 and Figure S1). The renal tube extended straight from the pericardial region toward the mantle collar, diagonally across the roof of the palial cavity and parallel to the pulmonary vein and renal vein. It was a straight tube, lined with a folded epithelium, white to white-yellowish, that tapered, smoothed and became transparent distalward until the level of the septum between pulmonary and hypopeplear cavities. In that place, it turned with an almost right-angle to form a short ureter (which lacked the two distinct flexures present in other close species) and finally opened behind the pneumostome. A very slender muscle thread appeared lengthwise on the ventral surface of the renal tube. A layer of very thin transverse parallel muscle fibers gave the inner side of the pulmonary wall a densely striated appearance (Figure 9 A,B).

The ovotestis showed a lobulate surface formed by several acini, with a collecting canal which continued into the ovispermiduct. The latter presented a short and very thin proximal segment emptying into the seminal vesicle, an expanded portion of bosselated surface, which narrowed down into a slender distal segment ending in the carrefour surrounded by the albumen gland, oviduct and spermiduct (Figure 9 C–F).

The voluminous albumen gland covered the carrefour and the origin of a bosselated, transverse tubular oviduct which followed a somewhat convolute course, described a nearly complete circle in contact with the albumen gland, and continued into a striated, oblong nidamental gland (Figure 9 C,D and Figure S1 A,B). The initial part of the nidamental gland increased in width to keep the diameter throughout its length or even slightly enlarged in its distal part, to subsequently narrow to give rise to a wrinkled-walled uterus followed by a short vagina. The latter shows a first conical narrowing part and a final short tubular part (without any bulbous swelling or sphincter-like thickening present in other close species) (Figure 9 E–G and Figure S1 A,B), opening into the female genital pore with a slightly thickened lip. All parts of the female reproductive system proximal to the vagina itself showed a pronounced width.

The yellowish spermatheca (Figure S1 A,B) had an oval body and gave rise to a thin, relatively long spermathecal duct which emerged laterally from the spermatheca instead of from its terminal extremity. The almost uniformly thin, smooth-walled spermathecal duct extended diagonally between the nidamental gland and the prostate until joining the final part of the uterus and the beginning of the vagina. The spermathecal duct was not dilated at its distal end (Figure 9 E–G and Figure S1 B).

Emerging from the carrefour, the spermiduct showed an initial slightly dilated part with a granular outer surface slendering into a uniformly thin, straight duct which ran on the ventral surface of the nidamental gland. The distal portion of the spermiduct widened to form a prostate of granular surface, relatively small size, narrower than the nidamental gland. The small, oblong, light grey prostate gland varied in shape from inverted-pear-like to elongated-potato-like, without ventral lengthwise fissure (Figure 9 E,F and Figure S1 C). In cross section, the prostate showed a simple, slit-like lumen (lacking internal folds known in other lymnaeids) (Figure 9 H and Figure S1 D). The prostate gave distally rise to a smooth-walled vas deferens which ran shortly in contact with the vagina and extended until looping caudalward. After this loop, the vas deferens became a uniformly thin, long duct to finally merge into a curved, penial sheath or phallotheca (Figure 9 E,F and Figure S1 E,F).

The penis sheath was regularly cylindrical, with a very slightly thicker proximal part (which showed no patent circlet of minute knobs or ring of papillae described in close species). The penis sheath was about a little bit more than half as long than the praeputium. The maximum width of the praeputium was around two or even three times thicker than the penis sheath. The shape of the praeputium was usually elongate-conical, gradually narrowing to terminate in the male genital pore, although it also appeared cylindrical thoughout most of its length and only tapering distally (Figure 9 E,F and Figure S1 E,F). This conical shape of the praeputium appeared to be an exception among the typical conical praeputium shape in Lymnaeidae. The length of the praeputium measured 0.73–1.96 mm (mean 1.13 mm) while that of the penis sheath was 0.36–0.94 mm (mean 0.72 mm), with a praeputium length/penis sheath length ratio of 1.20–2.23 (mean 1.60).

The radula was characterised by a tricuspid central tooth, presenting the large central cusp, a mid-sized right cusp and a small left cusp. The first lateral teeth were usually bicuspid, with two large cusps. In Mexico and also Iran there was a faint tendency to display a small associated denticle appearing at the inner border of the endocone, thus appearing tricuspid. The subsequent four to six lateral teeth were invariably bicuspid, the intermediate teeth were tricuspid, and the marginal ones multicuspidate.

#### Egg clusters

**Figure 10:**
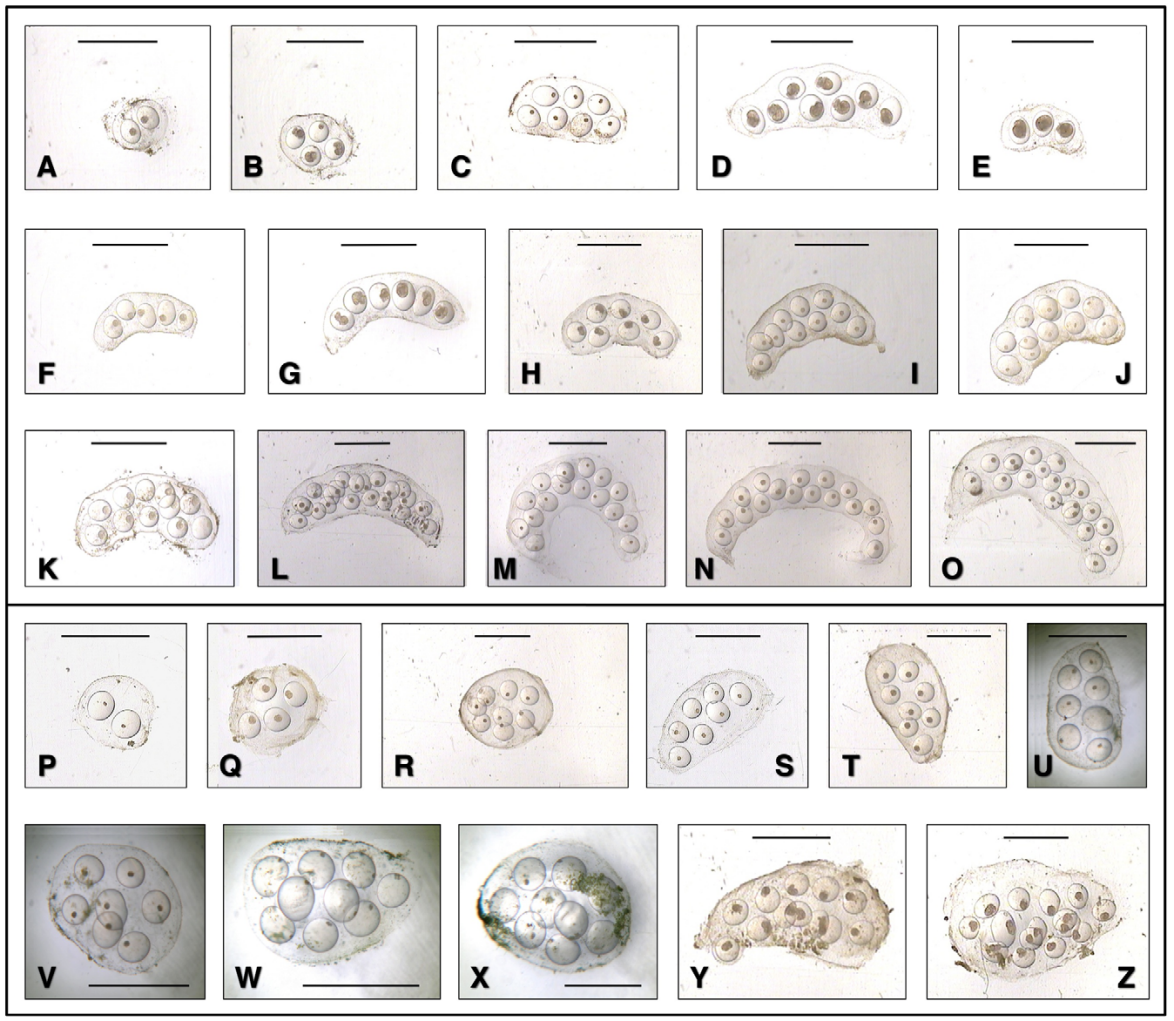
Gradual evolution of egg cluster lays in experimentally raised *Lymnaea schirazensis* and *Galba truncatula*: A–O) L. schirazensis: note trend to kidney shape in early lays (E–K) and final trend to banana-shape in late trends (L–O); only in very early lays, when shape is still round-oval (A, B) and may sometimes become elongate (C, D), it can be confused with clusters of G. truncatula. P–Z) *G. truncatula*: note general trend to round-oval shape (P–R, V–X) and occasional variation to elongate shape (S–U, Y, Z). Materials of *L. schirazensis* from strains originally collected in Xalpatlaco, Mexico (A, B, F, I, J), Jiutepec, Mexico (C), Albufera of Valencia, Spain (D, E, G, L, O), Tiba, Egypt (H), Escuela Obrego, Mexico (K). Materials of *G. truncatula* from strains originally collected in Qued Tiout, Marrakesh, Marocco (P–T, Y, Z) and Albufera of Valencia, Spain (U–X). Scale bar = 2 mm.

Shape variability of egg clusters and eggs was studied (Figure 10), and measurements and calculated ratios were noted (Table 5). Egg clusters were saccular, transparent, with a very thin outer membranous wall. The shape of these egg clusters showed an evident trend from kidney- to banana-like; the more curved and relatively more elongated and narrow the more numerous were the eggs inside. Small clusters containing only very few eggs (2–5) appeared rounded to oval, but the elongate-curved shape became soon apparent when the egg number increased. Eggs were from spherical to slightly ovoid, with a uniformly thick outer shell, usually appearing well separated from each other.

**Table 5:** Egg cluster and egg measurement comparison between different experimentally-maintained populations of *Lymnaea schirazensis* and *Galba truncaula* from different geographical origins.

	Clusters							Eggs		
Species and populations	Area (CA)	Roundness (CR)	Length (CL)	Maximum Width (CW)	CL/CW ratio	No. of eggs/cluster (CEN)	CEN/CA ratio	Length (EL)	Width (EW)	EL/EW ratio
***Lymnaea schirazensis*** **:**										
Tiba, Delengate, Egypt (n = 30 clusters+189 eggs)	4.77−13.27 8.31±2.14)	1.46−3.63 (2.22±0.53)	3.34−6.88 (4.74±0.94)	1.96−4.33 (3.01±0.65)	1.08−2.52 (1.61±0.32)	6−19 (11.50±3.30)	1.02−2.10 (1.40±0.28)	0.62−0.87 (0.72±0.04)	0.53−0.72 (0.61±0.03)	1.08−1.41 (1.18±0.06)
Albufera, Valencia, Spain (n = 30 clusters+195 eggs)	4.55−12.72 (7.77±1.89	1.23−2.21 (1.70±0.23)	2.81−6.07 (4.33±0.70)	1.94−3.78 (2.61±0.49)	1.19−2.14 (1.68±0.26)	6−23 (14.00±4.63)	1.32−2.48 (1.78±0.27)	0.63−0.82 (0.72±0.04)	0.52−0.65 (0.59±0.03)	1.11−1.35 (1.21±0.05)
Laguna, Atlixco, Mexico (n = 30 clusters+130 eggs)	1.80−6.49 (3.87±1.33)	1.31−2.08 (1.55±0.17)	2.07−4.47 (3.10±0.61)	1.19−2.46 (1.71±0.36)	1.43−2.28 (1.83±0.23)	3−12 (7.07±3.00)	1.29−2.19 (1.78±0.25)	0.57−0.71 (0.65±0.03	0.46−0.59 (0.53±0.02)	1.12−1.35 (1.23±0.06)
Esc. Obregon, Trinidad Tep., Mexico (n = 30 clusters+92 eggs)	1.47−6.33 (3.69±1.29)	1.22−1.63 (1.42±0.12)	1.73−4.21 (2.96±0.61)	1.01−2.03 (1.62±0.28)	1.47−2.42 (1.83±0.22)	2−10 (5.87±2.13)	1.11−2.02 (1.59±0.25)	0.60−0.83 (0.67±0.03)	0.48−0.65 (0.59±0.02)	1.11−1.36 (1.21±0.06)
Xalpatlaco 1, Atlixco, Mexico (n = 30 clusters+98 eggs)	1.34−7.22 (4.22±1.80)	1.13−1.76 (1.38±0.12)	1.72−4.19 (3.01±0.75)	1.02−2.48 (1.76±0.38)	1.33−2.05 (1.70±0.19)	2−10 (6.67±2.37)	1.11−2.24 (1.64±0.32)	0.56−0.83 (0.69±0.07)	0.46−0.69 (0.56±0.06)	1.12−1.40 (1.23±0.06)
Xalpatlaco 2, Atlixco, Mexico (n = 30 clusters+136 eggs)	2.04−7.49 (4.79±1.50)	1.25−2.05 (1.52±0.18)	2.02−4.41 (3.33±0.62)	1.19−2.92 (1.94±0.41)	1.39−2.02 (1.73±0.18)	3−18 (8.60±3.29)	1.00−2.40 (1.78±0.30)	0.58−0.79 (0.65±0.04)	0.48−0.68 (0.54±0.03)	1.12−1.35 (1.21±0.05
Jiutepec, Morelos, Mexico (n = 30 clusters+106 eggs)	1.67−6.77 (4.54±1.47)	1.01−2.34 (1.43±0.31)	1.75−4.15 (3.21±0.64)	1.20−2.90 (1.87±0.35)	1.23−2.29 (1.72±0.22)	2−10 (6.13±1.93)	1.03−2.29 (1.39±0.36)	0.61−0.86 (0.74±0.07)	0.50−0.69 (0.59±0.06)	1.14−1.49 (1.25±0.07)
TOTAL	1.34−13.27 (5.31±2.41)	1.01−3.63 (1.60±0.39)	1.72−6.88 (3.53±0.96)	1.01−4.33 (2.07±0.65)	1.08−2.52 (1.71±0.26)	2−23 (8.55±4.17)	1.00−2.48 (1.62±0.33)	0.56−0.87 (0.69±0.06)	0.46−0.72 (0.57±0.05)	1.08−1.49 (1.22±0.06)
***Galba truncatula*** **:**										
Albufera, Valencia, Spain (n = 15 clusters+92 eggs)	2.35−6.54 (4.58±1.29)	1.08−1.30 (1.18±0.06)	1.84−3.69 (2.73±0.53)	1.58−2.48 (2.11±0.26)	1.10−1.66 (1.29±0.18)	2−9 (6.13±2.23)	0.83−1.74 (1.31±0.21)	0.55−0.93 (0.71±0.07)	0.51−0.73 (0.60±0.04)	1.03−1.53 (1.18±0.10)
Oued Tiout, Essaouira, Marruecos (n = 42 clusters+100 eggs)	3.82−11.67 (6.45±1.48)	1.00−1.82 (1.24±0.15)	2.42−4.79 (3.39±0.60)	1.90−3.27 (2.43±0.29)	1.03−2.17 (1.45±0.28)	5−15 (7.81±2.37)	0.81−1.84 (1.21±0.24)	0.62−0.87 (0.75±0.05)	0.52−0.77 (0.62±0.05)	1.09−1.32 (1.20±0.05)
TOTAL	2.35−11.67 (5.95±1.64)	1.00−1.82 (1.22±0.13)	1.84−4.79 (3.29±0.67)	1.58−3.27 (2.34±0.32)	1.03−2.17 (1.41±0.26)	2−15 (7.37±2.43)	0.81−1.84 (1.24±0.23)	0.55−0.93 (0.73±0.06)	0.51−0.77 (0.61±0.05)	1.03−1.53 (1.19±0.08)

Range include minimum and maximum extremes, with mean±standard deviation SD in parentheses. Measurements in mm (area in mm^2^). n = number of specimens measured.

### Habitat

The ecological characteristics of *L. schirazensis* appeared to be very peculiar, mainly due to three aspects: (i) its marked amphibious characteristics, (ii) its frequency in antropophilic habitats, and (iii) the wide range of altitudes at which it is present.

Its pronounced terrestrial behaviour was surprising. In the laboratory, snails of this species appeared crawling on or attached to the lateral walls of the containers outside water. When taken with forceps and forced deep into water, they quickly moved out of it again.

**Figure 11:**
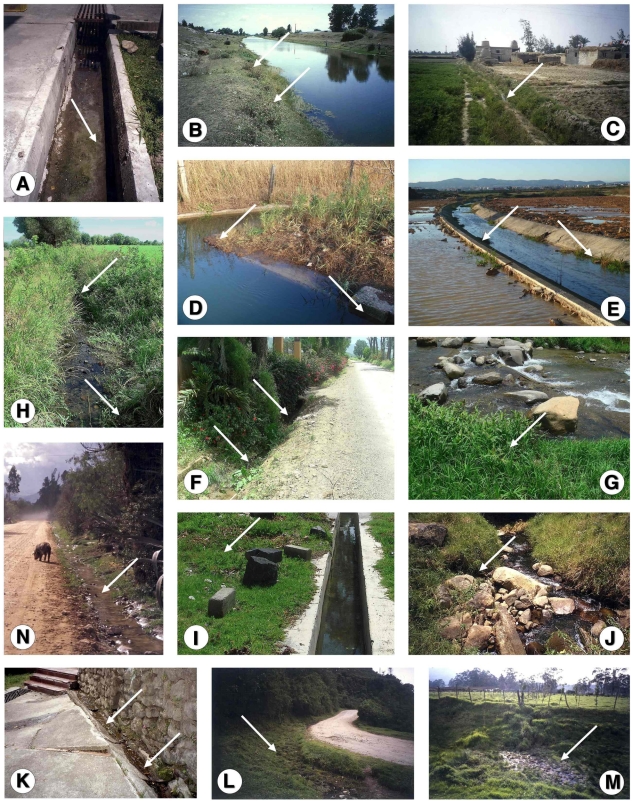
Environments of localities where *Lymnaea schirazensis* was collected: A) Garden of the Medicine Faculty, Rasht, Gilan province, Iran; B) Taleb Abad river, Bandar Anzali, Iran; C) El Kazza, Behera governorate, Egypt; D) Nules-Moncofar, Castellon province, Spain; E) Albufera of Valencia, Valencia province, Spain; F) Constanza, Departamento de La Vega, the Dominican Republic; G) Río Grande, Constanza, Departamento de La Vega, the Dominican Republic; H) Xalpatlaco, Atlixco, Mexico; I) Escuela A. Obrego, La Trinidad Tepango, Atlixco, Puebla, Mexico; J) Laguna de Fe y Alegría, El Valle, Merida, Venezuela; K) Hotel Valle Grande, El Valle, Merida, Venezuela; L) Guarandauco, Chillogallo, Ecuador; M) Machachi, Santo Domingo, Ecuador; N) Baños del Inca, Cajamarca, Peru.

In nature, this species was almost never found in water, nor even close to water edges, sometimes up to 1 m or farther from it (Figure 11 I). Outside water, it was usually found on the soil surface under grass besides slow running large rivers (Figure 11 B) or even under relative high bushes completely away from sunhine (Figure 11 H). The smallest amount of water to keep humidity was sufficient for this species to maintain populations, as in ditches of rural paths and dirt tracks (Figure 11 L,N) or even artificial cement canals in gardens (Figure 11 A) and drainage canalizations around buildings and dwellings (Figure 11 K). Man-made small irrigation canals around plant cultures also appeared to offer appropriate conditions for its development (Figure 11 C,D), as well as large drainage cement canals with almost no vegetation (Figure 11 E). Worth mentioning was its presence on mud and livestock footprints in and around animal farms (Figure 11 M).

Besides this link to antropophilic habitats and livestock-inhabited places, it was also found, however, in more silvatic habitats. Examples were specimens under the dense vegetation of the edges of fast running waters in mountain rivers and streams, where populations undoubtedly derived from dragging and floods by the water from upper areas (Figure 11 F,J,G).

Its distribution from lowlands to highlands was also worth mentioning. Thus, it was present below sea level, e.g. −23 m at the river edge near the Taleb Abad river mouth at the Caspian Sea, near Bandar Anzali city, in the northern Gilan province of Iran, or the very low altitude of localities such as El Kazza in the Nile Delta region of Egypt, the Mediterranean coast between the villages of Nules and Moncofar in Castellon province of Spain, and Rio Lurin, near Lima, Peru. On the other extreme, this lymnaeid also inhabited high altitude areas such as the 2,821 m of La Buena Esperanza, Cayambe, the 3,158 m of Guarandauco, Chillogallo, both in Ecuador, or the 2,611 m of Baños del Inca, Cajamarca, Peru. Intermediate altitudes were those of the 1,184 m altitudes around Constanza city in the central mountains of the Dominican Republic, those around 1,770–1,950 m of the localities close to Puebla in Mexico, and the 1,800–2,170 m range along the El Valle valley in Merida, Venezuela.

Interestingly, in none of the aforementioned geographical zones did this snail species appear to be the only lymnaeid present in the area. Its populations may appear mixed or close to populations of other lymnaeids (its smaller size may be a competing handicap with regard to other species). Accompanying lymnaeids were other morphologically and ecologically very similar species of the *Galba*/*Fossaria* group inhabiting the same place or water body, or neighbouring localities within the same fascioliasis endemic areas. Thus, *Galba truncatula* was found in all the areas studied in the Old World (Iran, Egypt, Spain). In the New World, *Lymnaea cubensis* shared the same areas in the Caribbean (the Dominican Republic) and Mexico, *L. humilis* in Mexico, and *G. truncatula*, *L. cubensis*, *L. cousini* and *L. neotropica* in South America (Venezuela, Ecuador, Peru).

Worth mentioning was that specimens of *L. schirazensis* sometimes appeared so mixed or close to one another with specimens of *G. truncatula*, that one was convinced to deal with a population of only one species, e.g. in Spain (Figure 11 D,E).

### Selfing and egg cluster laying

Egg cluster laying was verified to occur in all of the specimens from different populations maintained individually isolated after hatching (Table 6). Age and size when laying began differed somewhat from one individual to another within a population, less in populations of the New World (Mexico) than in those of the Old World (Egypt, Spain). Despite range overlap, those from Mexico proved to be markedly sexually precocious in age when compared to those from Egypt and Spain. Snail size when laying began did not show a similar correlation, suggesting that development speed and reaching a sufficient size may be more important for sexual activity.

**Table 6:** Characteristics of the first and five first egg clusters laid by experimentally-maintained *Lymnaea schirazensis* specimens of four populations from different geographical origins, followed up from the day of their individual isolation immediately after hatching.

	SNAILS		CLUSTER	CLUSTER CHARACTERISTICS						
POPULATIONS	Age (prelaying period in days)*	Size (height)**	Order No.	Area (CA)	Roundness (CR)	Length (CL)	Maximum Width (CW)	CL/CW ratio	No. of eggs/cluster (CEN)	CEN/CA ratio
Tiba, Delengate, Egypt (n = 10 snails)	35−50 (44.30±5.54)	2.84−3.91 (3.45±0.30)	1st	3.79−7.98 (5.94±1.47)	1.26−2.62 (1.93±0.46)	2.74−4.77 (3.79±0.66)	1.83−2.91 (2.41±0.28)	1.09−1.75 (1.58±0.22)	2−12 (6.40±3.17)	0.44−2.06 (1.08±0.50)
			1st to 5th	0.69−10.91 (4.43±2.31)	1.26−4.38 (2.15±0.67)	1.67−4.92 (3.25±0.86)	0.66−3.23 (2.08±0.67)	1.09−2.62 (1.64±0.36)	1−14 (5.23±3.33)	0.39−6.04 (1.35±0.05)
Albufera, Valencia, Spain (n = 10 snails)	25−44 (37.40±6.48)	4.46−5.82 (5.05±0.41)	1st	3.93−7.11 (5.13±1.24)	1.24−2.36 (1.52±0.37)	2.87−4.69 (3.44±0.59)	1.64−2.40 (1.99±0.25)	1.54−2.09 (1.73±0.16)	2−10 (7.40±2.76)	0.50−2.35 (1.45±0.53)
			1st to 5th	2.89−8.10 (5.00±1.29)	1.16−2.36 (1.45±0.21)	2.34−5.09 (3.38±0.56)	1.49−2.42 (1.95±0.23)	1.27−2.10 (1.73±0.16)	2−17 (8.91±2.87)	0.50−3.68 (1.86±0.68)
Esc. Obregon, Trinidad Tep., Mexico (n = 10 snails)	33−41 (35.40±2.67)	3.21−4.33 (4.01±0.31)	1st	1.08−3.73 (2.22±0.84)	1.24−1.71 (1.39±0.13)	1.52−3.07 (2.17±0.49)	0.91−1.64 (1.31±0.21)	1.34−1.94 (1.65±0.18)	4−10 (6.50±1.84)	2.15−5.54 (3.18±1.21)
			1st to 5th	1.08−8.93 (3.35±1.43)	1.19−1.92 (1.37±0.14)	1.52−4.45 (2.68±0.60)	0.91−2.61 (1.60±0.31)	1.34−2.07 (1.68±0.17)	2−10 (6.32±1.66)	0.90−5.54 (2.12±0.93)
Jiutepec, Morelos, Mexico (n = 10 snails)	27−35 (30.80±2.44)	4.51−5.69 (5.04±0.44)	1st	3.14−7.33 (5.08±1.50)	1.31−2.86 (2.09±0.56)	2.44−5.43 (3.73±0.91)	1.58−3.17 (2.18±0.45)	1.21−2.39 (1.74±0.43)	5−13 (9.40±2.88)	1.21−2.64 (1.89±0.50)
			1st to 5th	1.33−9.08 (4.91±1.88)	1.21−3.55 (1.72±0.53)	1.62−5.43 (3.50±0.89)	1.03−3.17 (2.01±0.49)	1.21−2.39 (1.75±0.27)	2−13 (8.66±2.83)	0.40−4.54 (1.97±0.86)
TOTAL(n = 40 snails)	25−50 (36.98±6.64)	2.84−5.82 (4.39±0.78)	1st	1.08−7.98 (4.59±1.89)	1.24−2.86 (1.73±0.49)	1.52−5.43 (3.28±0.93)	0.91−3.17 (1.97±0.51)	1.09−2.39 (1.67±0.27)	2−13 (7.43±2.87)	0.44−5.54 (1.90±1.08)
			1st to 5th	0.69−10.91 (4.40±1.84)	1.16−4.38 (1.63±0.51)	1.52−5.43 (3.18±0.79)	0.66−3.23 (1.89±0.47)	1.09−2.62 (1.70±0.24)	1−17 (7.38±3.06)	0.39−6.04 (1.86±0.91)

Cluster measurement range include minimum and maximum extremes, with mean±standard deviation SD in parentheses. Measurements in mm (area in mm^2^). n = number of specimens measured; * Days elapsed from day of hatching to day when first cluster was laid; ** Length of snail in the day it laid the first cluster.

Comparison of the measurements of the first and first five clusters laid by each individual snail also showed that slight differences may be found according to the populations. Results indicated that there is no gradual increase in cluster size nor in the number of eggs per cluster within the laying of a snail specimen. Thus, sometimes the first egg cluster laid by a snail individual was larger and contained more eggs than those laid afterwards (2nd to fifth clusters) by the same specimen (Table 6). However, somewhat larger clusters including a slightly larger number of eggs/cluster were laid by older and larger snail specimens (up to 20 and 22 eggs/cluster in specimens from Egypt and Spain, respectively).

**Figure 12:**
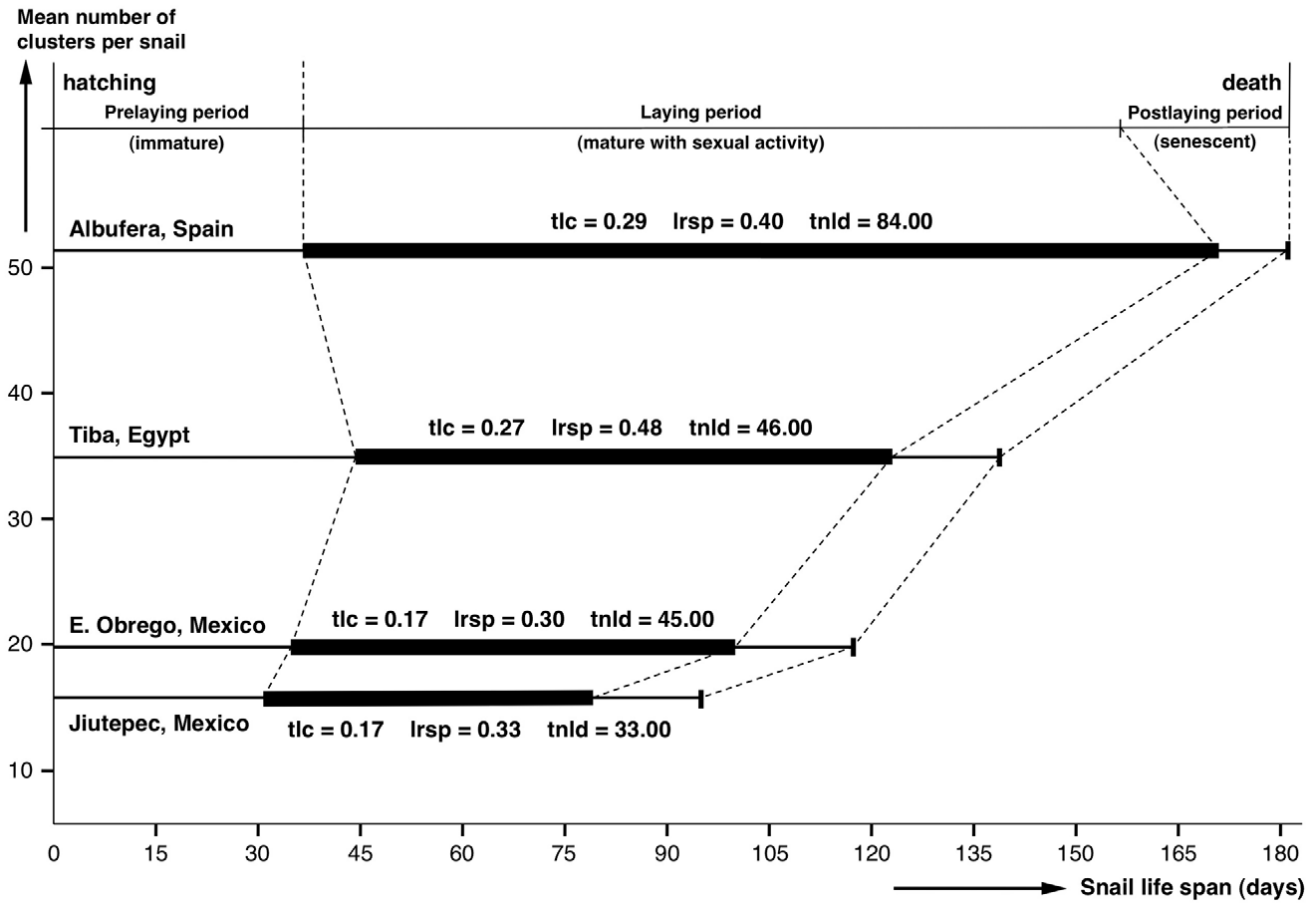
Comparison showing interpopulational differences of mean characteristics of life span and selfing reproduction capacity of isolatelly-maintained *Lymnaea schirazensis* specimens of four populations from different geographical origins, experimentally followed up from the day of their individual isolation immediately after hatching. Populations studied: Albufera of Valencia, Valencia, Spain; Tiba, Delengate district, Egypt; Escuela A. Obrego, La Trinidad Tepango, Atlixco, Puebla, Mexico; and Jiutepec, Morelos, Huauchinango, Mexico. n = 10 snail specimens followed per population (for details on intrapopulational variability ranges during the laying period, see Table 6 and Table 7); tlc = mean total laying capacity (number of clusters/life span in days); lrsp = mean laying rate in sexually active period (number of clusters/laying period in days); tnld = mean total non-laying days within laying period.

Snail life span was relatively short, of around 2.5–6.8 months. It includes a sexually active period of cluster laying lasting a total of 39–161 days, sometimes even only very few days before death. However, the final postlaying period may reach slightly more than 1.5 months in given specimens (Table 6 and Table 7; Figure 12). Both life span and laying period appeared to be longer in Old World (Egypt, Spain) specimens than in the New World (Mexico) specimens.

**Table 7:** Life span, selfing reproduction capacity and egg-cluster laying characteristics of isolatelly-maintained *Lymnaea schirazensis* specimens of four populations from different geographical origins, experimentally followed up from the day of their individual isolation immediately after hatching.

POPULATIONS	Snail life span* (days)	Laying period** (days)	Number of clusters laid per snail	Total laying capacity (No. clusters/life span in days)	Laying rate in sexual period (No. clusters/laying period in days)	No. of days in which only one cluster was laid	No. of days in which two clusters were laid	Total non-laying days within laying period	Max. No.of non-laying days between two laying days	Postlaying period*** (days)
Tiba, Delengate, Egypt (n = 10 snails)	102−198 (138.80±33.88)	51−136 (78.90±26.61)	23−54 (34.80±10.18)	0.12−0.45 (0.27±0.11)	0.21−0.72 (0.48±0.18)	23−46 (32.00±7.00)	0−4 (1.50±1.65)	23−101 (46.00±28.00)	2−36 (12.00±12.00)	6−37 (17.00±11.00)
Albufera, Valencia Spain (n = 10 snails)	157−205 (181.40±16.59)	100−161 (133.70±20.20)	38−70 (51.50±12.62)	0.20−0.43 (0.29±0.07)	0.26−0.60 (0.40±0.12)	37−66 (48.00±10.00)	0−7 (1.90±2.02)	49−112 (84.00±23.00)	6−34 (17.00±11.00)	1−33 (12.00±10.00)
Esc.Obregon, Trinidad T. Mexico (n = 10 snails)	88−139 (117.80±18.60)	45−90 (65.00±16.15)	9−35 (19.90±7.69)	0.10−0.25 (0.17±0.04)	0.20−0.42 (0.30±0.08)	9−35 (19.90±7.69)	0−0 (0±0)	34−69 (45.00±12.00)	6−24 (15.00±7.00)	3−37 (18.00±11.00)
Jiutepec, Morelos Mexico (n = 10 snails)	75−125 (94.80±16.02)	39−69 (48.50±8.61)	13−26 (16.20±3.65)	0.13−0.22 (0.17±0.02)	0.29−0.38 (0.33±0.03)	13−26 (16.00±4.00)	0−1 (0.20±0.42)	25−43 (33.00±5.00)	5−11 (8.00±2.00)	7–49 (17.00±12.00)
TOTAL (n = 40 snails)	75−205 (133.20±38.86)	39−161 (81.53±37.21)	9−70 (30.60±16.63)	0.10−0.45 (0.22±0.09)	0.20−0.72 (0.38±0.13)	9−66 (28.8±14.5)	0−7 (0.90±1.52)	23−112 (51.83±27.00)	2−36 (12.90±9.06)	1−49 (16.03±11.01)

Range include minimum and maximum extremes, with mean±standard deviation SD in parentheses. n = number of specimens measured; * life span = days elapsed from day of hatching up to day of death; ** laying period = days of sexual activity elapsed from first to last days when clusters were laid by the snail specimen, inclusive; *** postlaying period = time elapsed between last laying day and death.

This correlated with the number of clusters laid per snail, which was pronouncedly higher in Old than in New World specimens. Similar differences between Old and New World specimens were observed in the total laying capacity and the laying rate in the sexually active period.

Usually, snails only laid one egg cluster on the days they laid. Two clusters were sometimes laid by a snail specimen in the same day, although this appeared to be very rare in Old World (Egypt, Spain) specimens and occurred almost never (only once) in the New World (Mexico) specimens. Throughout the laying period, the number of days when no cluster was laid was larger in Old World (Egypt, Spain) specimens than in New World (Mexico) specimens. This fact seemed to be mainly related to the longer laying period in Old World specimens, as the differences in the maximum number of non-laying days between two laying days when comparing Old and New World specimens were too small (Table 7; Figure 12).

### Natural infection studies and experimental transmission assays

Along the ten-year study period, none of the 8,572 snail specimens from the 20 localities of the 8 countries belonging to this lymnaeid species proved to be naturally infected with fasciolid larval stages. This is the opposite of what happened with other lymnaeid vector species also collected in the same endemic areas. In other species, naturally infected specimens were detected through cercarial shedding even despite the usual very low snail infection prevalences.

Several experimental infection assays including a total of 338 snail specimens from different geographical origins with miracidia from experimentally-reared strains of *F. hepatica* and *F. gigantica*, also from different geographical origins, did not allow for successful fluke larval development. None of the infected specimens appeared to become parasitised nor reached cercarial shedding (Table 8). Only in two specimens from Albufera of Valencia, Spain, infected with miracidia of *F. hepatica* from Poland, could a few rediae be observed by transparency through the snail shell at 23 dpi, although neither shed cercariae thereafter. In a subsequent experiment with the same snail- and fluke-strains for verification, no infection and not even rediae were obtained in spite of the higher number of lymnaeid specimens infected.

**Table 8:** Characteristics and results of experimental infection assays of *Lymnaea schirazensis* from different geographical origins with miracidia from experimentally-reared strains of *Fasciola hepatica* and *F. gigantica* also from different geographical origins.

Experiment No.	Snail strain origin	Infected snail No.	Size of snails infected (height in mm)	*Fasciola* species used in infection assay	*Fasciola* strain origin	Miracidial dose (No.)	No. snails surviving at 30 dpi* (%)	Postinfection survival** (days)	Infection results
1	El Kazza, Egypt	30	3.5−5.0	*F. hepatica*	Pachago, Peru	1	26 (86.67%)	9−152 (81.4)	no snail infected
2	Valencia Albufera, Spain	65	3.5−6.5	*F. hepatica*	Hosh Essa, Egypt	1	6 (9.23%)	6−70 (17.2)	no snail infected
3	Valencia Albufera, Spain	30	3.5−6.0	*F. gigantica*	Gizza, Egypt	1	0 (0%)	9−29 (13.0)	no snail infected
4	Valencia Albufera, Spain	117	5.0−7.5	*F. gigantica*	Gizza, Egypt	1	0 (0%)	9 (9.0)	no snail infected
5	Valencia Albufera, Spain	10	4.5−6.0	*F. hepatica*	Bialowieza, Poland	1	6 (60%)	23−91 (60.7)	2 snails showing few rediae*** at 23 dpi*
6	Valencia Albufera, Spain	56	4.0−6.5	*F. hepatica*	Bialowieza, Poland	1	54 (96.4%)	30−111 (65.8)	no snail infected
7	Valencia Albufera, Spain	10	4.6−5.4	*F. hepatica*	Cajamarca, Peru	3	0 (0%)	17−30 (25.1)	no snail infected
8	Tiba Delengate, Egypt	20	3.6−6.1	*F. gigantica*	Quy Nhon, Vietnam	1	14 (70.0%)	14−78 (37.7)	no snail infected

*dpi = days postinfection; ** = range include minimum and maximum extremes, with mean in parentheses; *** = rediae in these two snail specimens never produced cercarial shedding.

Results on survivorship of snails after infection showed pronounced differences according to snail strains and fluke species and strains used. There were experiments in which no snail infected by either *F. hepatica* or *F. gigantica* was able to survive after 30 dpi, up to other experiments in which snails survived for more than 100 or even 150 dpi, thus suggesting no negative effects. Nonetheless, results suggested a lower survival rate in infections by *F. gigantica* than by *F. hepatica*. Furthermore, in *F. hepatica* infection assays, a faster and higher snail mortality rate appeared when using trimiracidial instead of monomiracidial infection (Table 8).

## Discussion

### Genetic distances

The twenty lymnaeid populations found in the eight countries of Asia, Africa, Europe, the Caribbean, North America and South America, show DNA sequences proving that all of them belong to the same species. The fact that this species presents an 18S rRNA sequence only is easily understandable given the slow evolution characteristics of this highly conserved gene [33], [34]. The low number of nucleotide differences at rDNA ITS-2 and ITS-1 level fits within the known intraspecific variability ranges of these two species markers [1], . However, it poses a question mark when considering the very wide geographical distribution (Figure 1), markedly variable habitats (Figure 11), and ecology (e.g., large altitudinal range) this lymnaeid species shows. Even the low number of only two and four haplotypes in the mtDNA 16S and *cox*1 gene fragments, respectively, including but a very few variable positions in both genes, is also surprising given the faster evolution of these mtDNA genes when compared to nuclear rDNA genes and spacers in invertebrates in general [46] and in lymnaeids in particular [45].

The interspecific comparison of the sequences of the snail species here in question demonstrates that it is a unique lymnaeid. It is genetically well different from *G. truncatula* and other species of the *Galba*/*Fossaria* group, as well as from stagnicolines and *P. columella*. At 18S rRNA level, *L. schirazensis* presents peculiar nucleotides in the helix E10−1 of the variable V2 area, which clearly differs from all other species (Figure 2). This fact in a well conserved gene such as 18S is worth mentioning [34]. At ITS-2 and ITS-1 level, the two main markers for species distinction [1], [16], [39], the very high number of nucleotide differences *L. schirazensis* shows when compared to all other lymnaeids must be highlighted. This is surprising when considering the pronounced morphological similarity of *L. schirazensis* with other lymnaeid species, mainly of the *Galba*/*Fossaria* group.

Concerning the sequences of the 16S and *cox*1 gene fragments, nucleotide differences between *L. schirazensis* and all other lymnaeids are also numerous (Figure 3 and Table S4), although less pronounced than those appearing in both ITSs despite the faster evolution of mtDNA. This may be related to the well known saturation of nucleotide positions in these two mitochondrial genes, a phenomenon previously described in Lymnaeidae [45].

The aforementioned surprising genetic distance results shown by the nuclear rDNA and mtDNA markers used can only be explained if the following conclusions are accepted:

the very high interspecific variation suggests an old evolutionary divergence of *L. schirazensis* from other lymnaeid species of the *Galba*/*Fossaria* group as well as from stagnicolines and *P. columella*;
the morphological similarity of *L. schirazensis* with other lymnaeid species of the *Galba*/*Fossaria* group indicates it to be a cryptic species by evolutionary convergence, probably as a consequence of adaptation to a similar amphibious way of life and similar habitats;
the very low intraspecific variability of *L. schirazensis* suggests a very recent worldwide spread from only one original strain and geographical source.

*Galba truncatula* is the lymnaeid species on which the largest number of studies has focused for decades, and one of the snail species with the largest body of literature and longer list of synonyms among molluscs in general. This very wide multidisciplinary body of literature is related to its applied importance as the main vector of *F. hepatica* throughout many countries [12], [29], in a life cycle which was the first to be elucidated among trematodes [72]. Thus, it is surprising that another widely distributed lymnaeid species, similar in morphology, biology and ecology, has always been confused with and kept masked under *G. truncatula* until now.

### Species ascription and specimen classification confusion

The very long list of species and varieties described long ago and later synonymised with *G. truncatula* [73] has been reviewed to analyse whether a species description published in the past could fit the characteristics of the lymnaeid species here in question. The older species name, fully fitting our material, appears to be *Lymnaea schirazensis*, described in 1862–1863 [71] from material collected in the locality of Shiraz, Iran, by von dem Busch who never published the description of his snail material (see Figure S2 and Figure S3). Consequently, the decision has been taken to ascribe the material described herein to the binomium *Lymnaea schirazensis* Küster, 1863. This species cannot be found in the literature published in recent decades. This ‘disappearance’ was undoubtedly due to its synonymy with *G. truncatula*, a proposal of Hubendick [73] apparently accepted by other specialists. *Lymnaea schirazensis* appears to have been given systematic validity and reported separately from *G. truncatula* only in Russia [74].

An exhaustive analysis of the old literature (see numerous references in [73]) suggests that several old species and variety names also synonymised with *G. truncatula* probably referred to *L. schirazensis* instead of to *G. truncatula*. The following items, in chronological order, are worth listing given the geographical locality the type materials came from:

the four *G. truncatula* varieties distinguished in Algeria [75] suggest that at least one of them could be the species here in question: *major* (not that of Moquin-Tandon) from Mostaghanem, *minutissima* from Alger, Géryville and Bône, *submalleata* from Djelfa, and *lanceolata* from Alger;
*L. persica* from the same type locality of Shiraz, Iran [76] is a synonym already previously proposed [77];
*L. hordeum* from River Euphrates, Mesopotamia [78];
*L. delaunayi* from Pasajes, northern Spain [79];
the variety *thiesseae* from Euboea, Greece [80] is very probably synonymous; this synonymy was already aptly discussed [81];
the two varieties *neapolitana* from Sebeto, near Naples, Italy [82] and *telouetensis* from Mount Atlas [83] merit further analysis.

The insufficient original description of *L. zrmanjae* from Zrmanja river, Dalmatian Croatia [84] may also lead to suspect a synonymy, although erroneously, as the latter indeed belongs to the family Hydrobiidae, as verified by the same author somewhat later (Brusina, 1902 in [85]) (see also Table S1).

These old names proposed in Europe and the Near East should be reviewed, but also more recent ones proposed for similar *Galba*/*Fossaria* lymnaeids in Europe, Asia and North America, so that further valuable information about the geographical distribution and worldwide spread of *L. schirazensis* can be obtained.

The coexistence with other very similar lymnaeid species of the *Galba*/*Fossaria* group within the same fascioliasis endemic areas, such as *G. truncatula* in the Old World, and *L. cubensis*, *L. humilis*, *L. cousini* and *L. neotropica* additionally to *G. truncatula* in the New World, suggests that a wider distribution of *L. schirazensis* may have been masked by lymnaeid specimen misclassifications. All of the aforementioned lymnaeids are vectors of fascioliasis and very easily confused when only classified from external characteristics or by non-expert scientists, mainly when specimens are small or mid-sized [15]. A size larger than the one of *L. schirazensis* is reached by *Galba truncatula* [29], *L. cubensis* [86], *L. humilis* [87], and *L. cousini* [88], [89]. Only *L. neotropica* is of a size similar to that of *L. schirazensis* [15]. *Galba truncatula* is present in the Gilan province of Iran [89], in the Nile Delta region [90], [91], along the Mediterranean coast of the Valencian Community, Spain [1], [92], and in Merida State of Venezuela [93]. *Lymnaea cubensis* is known throughout Hispaniola, including both the Dominican Republic [94] and Haiti [95], Puebla province of Mexico [96] and Merida State of Venezuela [93], [97]. *Lymnaea humilis* is the species most frequently mentioned to be involved in fascioliasis transmission in Mexico (see review in [45]), sometimes under the names of *L. modicella* and *L. obrussa* [28], [98], both synonymised with *L. humilis* [73], and also reported in Puebla State [96]. *Lymnaea cousini* has been reported in fascioliasis endemic Andean areas of Ecuador [55], [73], [99], and also in Merida State of Venezuela [88], [93]. However, this Venezuelan material later proved to belong to another species, namely *L. meridensis* [55]. *Lymnaea neotropica* has been reported, under the name of *L. viatrix* [15], in several fascioliasis endemic Andean valleys of Peru, as is the case of Cajamarca [100], [101].

### Genotypic and phenotypic differentiation between *L. schirazensis* and *G. truncatula*

Henceforth, when DNA sequencing techniques are used, the molecular differentiation of *L. schirazensis* and *G. truncatula* can be easily made by comparison with the following sequences already available at GenBank/EMBL:

*L. schirazensis*: specific classification can be based on the nucleotide sequences of ITS-2 rDNA, ITS-1 rDNA, mtDNA 16S, and mtDNA *cox*1; for supraspecific classification, the nucleotide sequences of the 18S rDNA and of both ITSs can be used (Table 1); the amino-acid sequence of the mtDNA COX1 protein does not appear to be helpful for any discrimination.
*G. truncatula*: specific classification can be based on the nucleotide sequences of ITS-2 rDNA [1], [15], [35]; ITS-1 rDNA [15], [35], [39], and mtDNA *cox*1 [15], [57]; for supraspecific classification, the nucleotide sequences of the 18S rDNA [33] and of both ITSs can be used; the mtDNA COX1 amino-acid sequences [15], [57] do not appear to be useful at any level.

**Figure 13:**
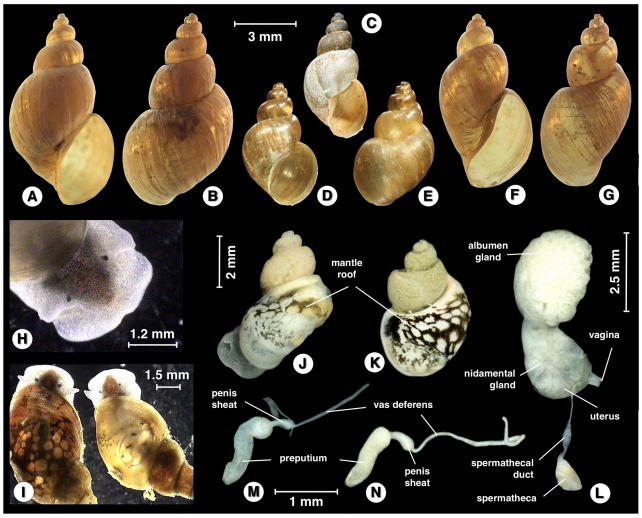
*Galba truncatula*: A–G) Shells in ventral and dorsal views of specimens from Albufera of Valencia in Spain (A,B: 9.33 mm high), Sachsen in Germany (C: 6.20 mm), Nules in Spain (D,E: 6.00 mm), and Qued Tiout in Morocco (F,G: 8.29 mm). H) Eyes and tentacles in living specimen. I) Comparison of living *L. schirazensis* (left) and *G. truncatula* (right) showing differences in (i) eyes, (ii) tentacles and (iii) mantle roof colour through the shell (by infralighting). J,K) Mantle roof in specimens from Nules, Spain (J) and Iran (K). L) Part of reproductive system in ventral view. M,N) Male terminal organs in specimens from Nules, Spain (M) and Iran (N). Scale bars: A−G = 3 mm; H = 1.2 mm; I = 1.5 mm; J,K = 2 mm; L = 2.5 mm; M,N = 1 mm.
Tentacles: of different form, being elongate, slender and with a narrow base in *L. schirazensis* (Figure 8 A–E), whereas they are wider and with a wide base in *G. truncatula* (Figure 13 G);
Eyes: of different size, being relatively big and markedly larger in *L. schirazensis* than in *G. truncatula* (Figure 13 H,I);
Egg clusters: of different shape trend, showing an evident trend from kidney- to banana-like, the more curved, elongated and narrow the more numerous are the eggs inside, in *L. schirazensis* (Figure 10 A–O); clusters tend to keep a rounded to oval shape even when containing more eggs, in *G. truncatula* (Figure 10 P–Z);
Egg number per cluster: although no significant differences were found between both species in our study, according to the literature it appears that a higher mean egg number per cluster of 12–15 may be the usual rule in *G. truncatula*, with exceptions of a lower number depending on the soils of the different areas and sometimes even on the season [29]; this mean number shows a lower level range of around 6–14 in *L. schirazensis* (Table 5);
Mantle colour: mantle roof from dark brown to blackish throughout, with unpigmented white-greyish round spots, this black pigmentation giving a dark appearance to the shell of living specimens by transparency in *L. schirazensis* (Figure 8 D–I); in *G. truncatula* the hypopeplear region of the mantle roof shows larger unpigmented whitish spots giving a pale appearance to the shell of living specimens by transparency (Figure 13 I–K);
Radula: first bilateral teeth bicuspid in most populations, although in given populations a small associated denticle appears thus becoming tricuspid in *L. schirazensis*, whereas it appears tricuspid in *G. truncatula*;
Male organs: with different praeputium length/penis sheath length ratio, which is of 1.20–2.23 (mean 1.60) in *L. schirazensis* (Figure 9 E,F and Figure S1 E,F); in *G. truncatula* the length of the praeputium is 0.82–2.80 mm (mean 1.41 mm) and that of the penis sheath is 0.22–0.64 mm (mean 0.37 mm), with a praeputium length/penis sheath length ratio of 2.50–5.90 (mean 3.44), according to data obtained by measuring specimens from Öland, (Sweden), Kühren, Ettenheim and Reusten (Germany), and Scharflinge (Austria) (Figure 13 M,N);
Ecology: with different water dependence, showing marked amphibious characteristics with a pronounced terrestrial behaviour in *L. schirazensis* (see habitat types in Figure 11 A–M), whereas aquatic inside freshwater borders or amphibious but more water-dependent in *G. truncatula*.

When DNA sequencing techniques are not available, to avoid further confusion between *L. schirazensis* and *G. truncatula* given the evident difficulty in distinguishing both species due to the morphological variability of their very similar shell, the following differential phenotypic characteristics ought to be considered:

Shell size: smaller in *L. schirazensis* (Table 4), reaching a shell length of up to 12.00 mm in *G. truncatula* [29], whereas it shows a maximum of only 8.06 mm in *L. schirazensis*; other shell characteristics may be helpful, such as the regularly convex whorls and straight columella in *L. schirazensis* (Figure 7 A–D) when compared to the stepped whorls and folded columella in *G. truncatula* (Figure 13 A–F); however, shell variability may easily give rise to confusion when based only on these aspects;

### Phylogenetic relationships and supraspecific classification

The phylogenetic analyses performed (Figure 5 A,B and Figure 6 A,B) confirms the ascription of *L. schirazensis* to the *Galba*/*Fossaria* group, as already suggested by the morphoanatomic characteristics of this species. Thus, its transfer to the genus *Radix* as proposed by some researchers (e.g., http://clade.ansp.org/malacology/collections/search.php?mode=browsetypes&targetfamily=Lymnaeinae) does not appear supported.

Unfortunately, there is great confusion around the generic names proposed for *truncatula* and proximal species in the Americas (the Central American *cubensis*, the North American *humilis* and *bulimoides*, and the South American *viatrix* and *diaphana*, only to mention the most important), all of which share the capacity of transmitting *F. hepatica* [1]. Besides the genus *Galba* Schrank, 1803 (type species: *truncatula*) [1], [14], [20], [34], [102], [103], in which it was included [50], other genera having frequently been used to include the New World *Galba*/*Fossaria* species, such as *cubensis* and *viatrix*, are: *Fossaria* Westerlund, 1885 (type species: *truncatula*) [104]–[107], *Nasonia* Baker, 1928 (type species: *cubensis*) (see [73]), the subgenus *Fossaria* (*Bakerilymnaea*) Weyrauch, 1964 (type species: *cubensis*) [104], [106], [108], and the subgenus *Lymnaea* (*Afrogalba*) Kruglov et Starobogatov, 1985 (type species: misassigned to *cubensis*; correct type species is *mweruensis* Connolly, 1929) [109].

In the many phylogenetic reconstruction assays performed, *L. schirazensis* appeared in three different positions:

In the same clade with *L. humilis, L. cousini* and *L. meridensis*: That situation was the one obtaining higher support values, appearing in a higher number of phylogenetic trees, and supported by both rDNA (ITSs) and mtDNA (16S−*cox*1) sets. This may support the classification of *L. schirazensis* within the genus *Pseudogalba* Baker, 1913 (new name proposed for *Simpsonia* Baker, 1911; type species: *humilis*) (see [73]). However, the node branching *L. schirazensis* together with *L. humilis*, *L. cousini* and *L. meridensis* never appeared with sufficiently high support values.
Basal to all other *Galba*/*Fossaria* members: This situation does not appear to fit any of the genera erected within *Galba/Fossaria* so far.
Clustering together with *G. truncatula*: This relationship supporting its inclusion in the genus *Galba* is the one appearing in fewer trees.

Consequently, prudence suggests to keep *L. schirazensis* within the genus *Lymnaea sensu lato* for the time being.

### Ecological adaptability, migration capacity and geographical distribution

Ecological characteristics of *L. schirazensis* are peculiar when compared to other Lymnaeidae. The amphibious behaviour differ between lymnaeid groups. *Radix* and stagnicoline species prefer permanent, larger and deeper water bodies, which they leave only sporadically. On the opposite, *Galba*/*Fossaria* species may be found on mud or wet sites very close to water in both temporary and permanent water bodies [1], [5]. This approaches *L. schirazensis* to *Galba*/*Fossaria*. However, no other lymnaeid shows such a preferential terrestrial behaviour.

Its amphibious characteristic and wide habitat range, mainly its presence in habitats with very small amounts of freshwater, suggest a great adaptation capacity. Additionally, its frequency in antropophilic and man-made habitats and its survival capacity outside water suggests that this snail can be passively transported by livestock and/or human activities, as demonstrated in *G. truncatula* [12], which shows similar habitat characteristics [29].

Selfing in *L. schirazensis* remembers the autofecundation preference in *G. truncatula* [29], [110]–[113] and other *Galba*/*Fossaria* species (Khoubbane et al., unpublished). Its laying capacity (Table 6 and Table 7) appears to be greater than in *G. truncatula* [29], with a higher number of clusters/month laid per specimen along the laying period. Both selfing and high laying capacity will undoubtedly facilitate its capacity to colonise neighbouring or distant places and expand geographically. Selfing, high egg laying, short life span, great spreading and colonisation capacity are typical of r-strategist organisms (see review on r/K selection in [114]).

This suggests that *L. schirazensis* may be far more widespread. Such a wider distribution may not only concern the eight countries noted, but also neighboring countries and others further north or south. However, studies are needed to assess whether its terrestrial life trend, outside water, may restrict its capacity to colonise colder areas, contrary to *G. truncatula* which inhabits high latitude regions [29], [73]. In Asia, *L. schirazensis* has been noted to have a distribution covering Iran, Afghanistan, mid-Asia and the Caucasus [74]. Although the geographical proximity suggests that it may be present further away from Iran, the more northern, colder latitudes indicate that a molecular verification of these Asian lymnaeids is indispensable.

### Phylogeography and continental spread during the historical postdomestication period

The wide distribution of *L. schirazensis* in different continents is bewildering. The very low variability in its DNA sequences indicate that such a distribution was reached relatively recently. Molluscs are known to be able to follow long-distance dispersal, transported by migrating birds but mainly by human activities. Certain freshwater snail vectors have been reported even very far away from their area of origin (e.g., different continents) [115], including examples of lymnaeids [12], [35], [36]. A few bird species cross the Atlantic Ocean [116], [117], but the probability of a lymnaeid transport by a bird is very low owing to the low number of bird individuals undertaking this journey every year. Yet, proof of an incredible transequatorial dispersal of snails by birds is there [118].

**Figure 14:**
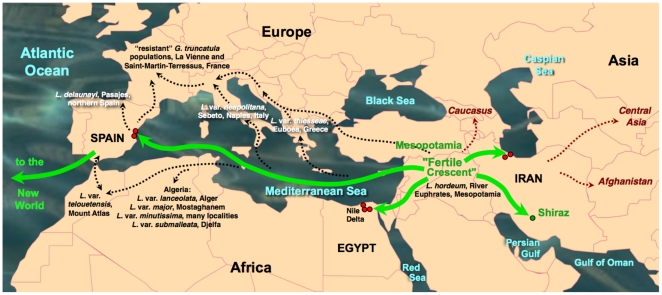
Old World spread of *Lymnaea schirazensis* combined haplotype ITS-2 H1-ITS-1 HA-16S HA-*cox*1 Ha during the livestock postdomestication 10,000-year period from the “Fertile Crescent” region of origin in the Near East, including expansion route into the New World from southern Spain during the early period of colonization about 500–400 years ago (green lines and red dots). For historical and archeological data supporting this recent spread, see text. Green dot of Shiraz, Iran = type locality of *L. schirazensis*; black lines = spreading routes into Europe and Africa to cover the geographical distribution suggested by the presumed species and variety synonyms (see text) and “resistant” *Galba truncatula* populations described in France [29]; brown lines = spreading routes into Asia according to the geographical distribution noted by Kruglov [50].

**Figure 15:**
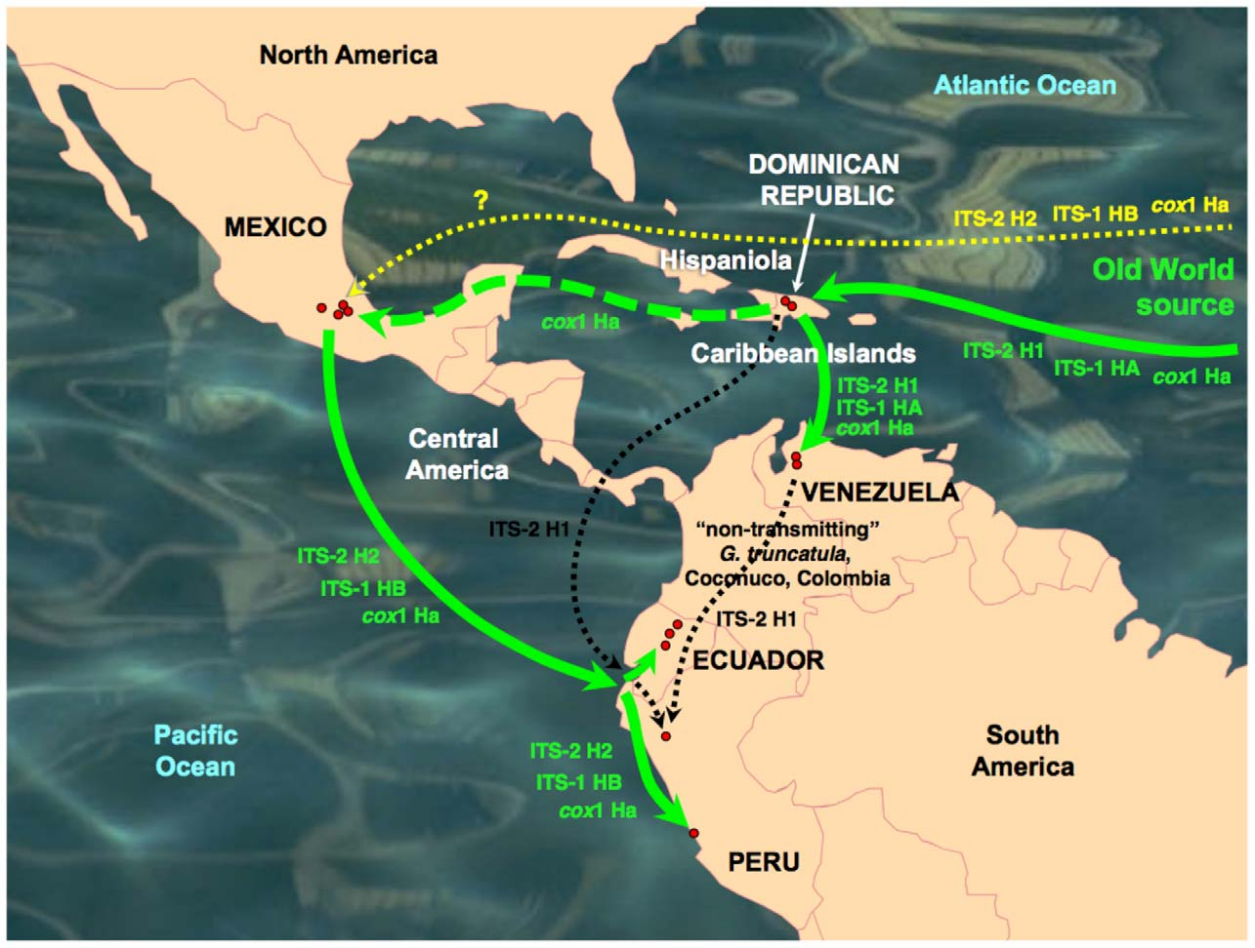
New World spread of the different combined haplotypes of *Lymnaea schirazensis* from remote Old World source(s) in Europe (only or mainly southern Spain) during the early period of colonization about 500–400 years ago (green lines and red dots), illustrating the dispersal role played by Hispaniola island. Combined haplotype ITS-2 H2-ITS-1 HB: (i) origin whether in Mexico itself after introduction from Hispaniola (green broken line) or from an unknown European source (yellow ? broken line), and (ii) subsequent introduction into Ecuador and Peru by a sea route from Mexico. Introduction of ITS-2 H1 in northern Peru whether by a maritime route through southern Central America (Panama isthmus) or by a terrestrial route from Venezuela (alternative dark broken lines). For historical and archeological data supporting this recent spread, and further details on mtDNA gene isolated haplotypes (16S HB and *cox*1 Hb, c and d), see text.

Evidence suggests that *G. truncatula* may remain in dried mud stuck to the feet of ruminants, then go into hibernation or estivation, and be able to reactivate once in a new location following contact with water or sufficient humidity [12]. Thus, livestock import/export appears related to the continental spread of *G. truncatula*. Results from our field and laboratory work suggest that *L. schirazensis* has spread following the same passive transport way of *G. truncatula*: (i) similarity of livestock-frequented habitats (usually found in mud poached by livestock hooves), (ii) long survival outside freshwater (allowing for long distance transport), (iii) selfing (facilitating invasion of new areas), and (iv) high and fast egg laying capacity (facilitating establishment in a new area). Hence, a review of human history, commercial routes and livestock import/export between the countries inhabited by *L. schirazensis* offers a likely way of understanding its recent spread (Figure 14 and Figure 15). This chronological spread shows a clear parallelism with the origin and spread of *F. hepatica* and also in part with the spread followed by *G. truncatula* [12].

#### Spreading origin in the Fertile Crescent

Historical evidence suggests that the original area from where *L. schirazensis* spread may have been in the Near East, around 10,000 years ago at the dawn of the Neolithic in the region known as the Fertile Crescent, including modern-day Israel, Jordan, Lebanon, western Syria, southeast Turkey, Iraq and western Iran [119]. This region was a major domestication center of livestock, mainly goats and sheep, but also cattle and pigs [120], [121]. The presence of *L. schirazensis* in Gilan, Iran, and the probable synonyms of *L. persica* from Shiraz, Iran [76], [77] and *L. hordeum* from River Euphrates, Mesopotamia [78] agree with such an origin (Figure 14). This Neolithic culture expanded geographically 6,400 years ago [12].

#### Old World spread into Africa, Europe and Asia

A first spreading of *L. schirazensis* probably occurred into northern Egypt together with sheep and goats from the 7th millenium BC [122], [123].

Its introduction into Spain may be related to the extensive use of a Mediterranean route by sea and/or along Mediterranean coastal regions [124]. The genetic diversity in Portuguese sheep suggests a flow from the Fertile Crescent to the Iberian Peninsula, occurred with the Phoenicians, Greeks and/or Romans [125], and also later in the 11th and 12th centuries, without forgetting the well-documented exchange between Iberia and the Maghreb during the Moslem period [124]. The high diversity of Iberian goat breeds [126] and cattle migration along the Mediterranean coast [127], further substantiate the importance of the Mediterranean in past livestock movements by connecting Iberia to the Near East, North Africa, and southern Europe [125], [128].

These trans-Mediterranean and circum-Mediterranean ancient livestock movements may also explain the likely presence of *L. schirazensis* in Greece, southern Italy, and northern Spain, as well as northwest Africa, namely in Algeria and also Morocco, if the probable synonyms here proposed are molecularly confirmed in the future. The quotations of *L. schirazensis* in Russia [74], if molecularly verified in the future, may be attributable to the ancient exchange between Asian and European sheep [129]. The Silk Road, active from around 138 years BC until the 15th century, connected eastern China with the Near East by three routes through Kirgistan, Tajikistan, Uzbekistan and Turkmenistan [130]. Camels, taurine and zebu cattle were mainly used for the transportation of goods and merchandise [131] (Figure 14).

#### Trans-Atlantic spread into the Americas

The spread of *L. schirazensis* into the Americas may have benefitted from livestock transported by the Spanish conquerors during the early colonisation period. Cattle and sheep were the species most transported, although goats were also included. Livestock continued to be exported from the Old World to the Americas during the 16th, 17th and 18th centuries taking advantage of an intense regular commercial exchange [12].

In the Caribbean, a founding population of approximately 300 cattle was first introduced in Hispaniola in 1493. Cattle mostly originated from Andalusia [132]. Sevilla, with its fluviatile port, was the point of ship departure, which suggests that *L. schirazensis* may be present in southern Spain, too. Hispaniola was used as base station for further expeditions [133]. Thus, livestock multiplied pronouncedly on Hispaniola and animals were taken from there and introduced first into Cuba, then Puerto Rico and later Jamaica [134]. This suggests that *L. schirazensis* may also be present on those large islands, confused with *L. cubensis* as in the Dominican Republic.

By 1525, cattle had already spread throughout much of Central and South America. A similar intense livestock introduction process followed to present-day Mexico, Venezuela and Peru. Livestock was initially imported into Mexico from the Caribbean, mainly Cuba [135] but later also directly from Spain [136]. Thus, Puebla became a thriving agricultural area from the early period [133]. However, South America was colonised “from the back” instead of directly from Europe [133], including first Peru, entering through the northern Tumbes and taking advantage of the rural Andean routes of the Inca people after a maritime route through Panama [133], [137]. Later, directly entering through the Caribbean Sea, it was Venezuela, where livestock multiplied in the Merida mountainous area in the 1600 s [138]. In the early colonialisation period, ship exchange activities between the Peruvian Tumbes area and Mexico and southern Central America took place.

Although a trans-Andean livestock introduction route was launched between western Andean Venezuela and Colombian Bogota and also further southward [139], the introduction of *L. schirazensis* with livestock and humans is very likely to have occurred from northern Peru. Livestock trade between Quito and Lima through Cajamarca became very intense at a certain period [133]. Nevertheless, the presence of *L. schirazensis* in Colombia should not be ruled out, as indeed the only published report of non-infected *G. truncatula* in Aguas Tibias, Purace municipality, Coconuco, may also be a misclassification [140] (Figure 15).

### Implications for fascioliasis

#### A *Galba*/*Fossaria* species unique due to its unsuitability for fasciolid transmission

In the fascioliasis endemic areas analysed, our studies demonstrated that the pronouncedly different susceptibility/resistance characteristics of *Galba*/*Fossaria* species, initially considered to be related to different strains of both lymnaeids and *F. hepatica*, were indeed due to the presence of a hitherto-overlooked lymnaeid species. This unnoticed species was mixed and confused with *G. truncatula* and other lymnaeids whose transmission capacity was experimentally assessed.

In these areas, a naturally-infected *L. schirazensis* specimen was never found, despite (i) inhabiting localities where high fascioliasis prevalences are known, and (ii) being present in habitats where *G. truncatula* and other *Galba*/*Fossaria* species are typically found to be infected (e.g., mud frequented by livestock). This absence of natural infection agrees with the total lack of susceptibility to laboratory infection by different geographical strains of *F. hepatica* and *F. gigantica*. Experimental assays have shown that fasciolid larval stages do not develop until cercarial shedding inside *L. schirazensis*. In several experiments, snails showed a short survival after infection, whereas in other assays the long post-infection survival of snails indicated no negative effects due to infection. These results were independent of snail age and size (Table 8).

When considering the infection incompatibility of *L. schirazensis*, its phylogenetic relationships are surprising (Figure 5 and Figure 6). The phylogenetic trees locate it inside the *Galba*/*Fossaria* group, whose members are all *F. hepatica* transmitters [15], as *L. humilis* and *L. cousini* [45], [55], with which it shares the same branch. The terrestrial trend of *L. schirazensis* may have evolutionarily been the original cause of susceptibility loss due to the very low fasciolid miracidium contact likelihood in a lymnaeid entering water only very sporadically.

#### Geographical distribution of *G. truncatula* and other similar *Galba*/*Fossaria* vector species

*Lymnaea schirazensis* has always been confused with *G. truncatula* in the Old World (with the exception of Russia and neighbouring countries, where its classifications should, however, be molecularly verified [74]) and with *G. truncatula* or other *Galba*/*Fossaria* species in the Americas. Hence, the distribution of these *F. hepatica* vectors [29], [73] should be re-assessed.

The strict *Fasciola* sp./lymnaeid sp. specificity [1] has allowed for the use of lymnaeid vectors as biomarkers of the distribution of the disease in humans and animals [16], [141]. Relationships between lymnaeid vector species and the different epidemiological scenarios and transmission patterns of fascioliasis are worth emphasizing [12]. Factors important for fascioliasis epidemiology and control such as type of water habitats, population dynamics, temperature thresholds, seasonality, or infection susceptibility differ depending on the different lymnaeid species. Fascioliasis forecast mathematical indices are based on lymnaeid-interacting climatic factors such as air temperature, rainfall/irrigation and/or potential evapotranspiration [7], [8], [142]–[144]. Forecasting by Remote Sensing (RS) and Geographical Information System (GIS) methods is based on a more complex suite of lymnaeid-interacting environmental factors, such as surface hydrology, vegetation indices and temperature data [13], [145]–[149]. Henceforth, when evaluating the local accurateness of mapping results, potential confusion of lymnaeid species ought to be verified. Such confusion throughout different zones inside an endemic area may explain local disease data which do not fit lymnaeid distribution maps. Similarly, the parallelism between the geographical distribution of *F. hepatica* and *G. truncatula* in low resolution maps is to be reassessed once more data on the distribution of *L. schirazensis* become available.

#### A new biomarker for the follow-up of livestock movements and fascioliasis spread

Genetic data, and paleontological, archeological and historical records, have shown a worldwide spread linking livestock (transportation, transhumance and trade of mainly cattle, sheep and goats), parasites (*F. hepatica* and *F. gigantica*), and lymnaeid vectors (*G. truncatula*) during the animal post-domestication period (last 10,000 years) [12]. Our results in field and laboratory studies suggest that *L. schirazensis* is another species having been involved in the same evolutionary framework. Its link with livestock movements indicates that *L. schirazensis* may be a good biomarker for the follow-up of fascioliasis spread. Fascioliasis introduction into the Dominican Republic, where *G. truncatula* is absent, is an example.

#### Fasciolid susceptibility/resistance in *G. truncatula* and other lymnaeid vectors

Trematode-snail specificity is a complex phenomenon related to infectivity, susceptibility, resistance, immunity, compatibility, host attraction, finding and recognition, phylogeny, and genetic variability [150]. In *F. hepatica* and *F. gigantica*, lymnaeid spectrum limits are not clear [1], [16]. Contradictory results have always been related to different susceptibilities of different allopatric populations [12].

When experimentally comparing different *G. truncatula* populations, all were susceptible to *F. hepatica* infection [26], [27], [151]. However, different infectivity rates appear regarding the same geographical strain of *F. hepatica*, even including marked snail inter- and intrapopulational differences within a reduced endemic area [152]. Similar results also appear in other lymnaeid species concerning both fasciolids [29], [30]. *Galba truncatula* populations never showing cercarial shedding in endemic zones and resistant to *F. hepatica* in the laboratory have been described [29]. Differences in miracidium infection capacity related to different geographical *F. hepatica* strains [153] and different definitive host species [29] have been discussed.

However, assumptions on *F. hepatica*/*G. truncatula* susceptibility/resistance in endemic areas might be erroneous due to overlooked confusion with *L. schirazensis*. In the field, misclasifications can be easily understood due to (i) morphological similarity, (ii) habitat resemblance, and (iii) even mixing of the two species within what appears to be only one population (as in Nules-Moncofar, Spain). In the laboratory, confusion is also easy because in *L. schirazensis* miracidium penetration is successful. Molecular and morphological tools allowing for the distinction of *L. schirazensis* have to be applied henceforth in susceptibility/resistance studies. The three “resistant” *G. truncatula* populations described in France [29], [154] are examples to be verified in that way.

#### A new laboratory model for studies on susceptibility/resistance in *F. hepatica*/lymnaeid interaction

Trematode/snail interactions is a research field with disease control applications. *Lymnaea schirazensis* assembles features to become a good model for studies on genomics and proteomics characteristics of resistance to fasciolid infection: A) host attraction, finding and recognition by the *F. hepatica* miracidium take place; B) miracidium penetration and a certain sporocyst-redial development occurs, opening for studies on larval development abortion; C) its genetic link with *Galba*/*Fossaria* suggests functional aspects and genetic basis of immunological response similar to those in the main *F. hepatica* vectors; D) amphibious resemblance with *Galba*/*Fossaria* members suggest physiological similarities with the main *F. hepatica* vectors; E) its laboratory rearing is easier than in *G. truncatula* and other *Galba*/*Fossaria* vectors, owing to: (i) easy adaptation to experimental conditions, (ii) sexual precocity, (iii) high fecundity, (iv) short adult life span, and (v) selfing permitting the launch of genetically pure laboratory strains; F) postinfection survival allows for reinfection assays to assess immunological sensitisation.

## Supporting Information

Figure S1 Photographs showing soft parts of *Lymnaea schirazensis*: A, B) part of reproductive system in ventral view (prostate removed in B); C) prostate and beginning of vas deferens; D) section of prostate showing absence of internal folds; E, F) male terminal organs.
(TIFF)

Figure S2 Original description of *Lymnaea schirazensis* by Küster in 1863 [71] according to snail materials collected by von dem Busch in the locality of Shiraz, Iran (described under species number 78, page 53, issue 184, year 1863).
(TIFF)

Figure S3 Original figures of *Lymnaea schirazensis* by Küster in 1862 [71] according to snail materials collected by von dem Busch in the locality of Shiraz, Iran (drawings in plate 11: figure numbers 28 and 29 showing natural size specimens and figures 30 and 31 showing enlarged specimen, in issue 182, year 1962).
(TIFF)

Table S1 **List of species (and subspecies) of molluscs and trematode parasites included in this study according to the zoological nomenclature, in alphabetical order according to names used in the text.** Clarification notes in brackets; *s.l.* = *sensu lato*.
(PDF)

Table S2 **Pairwise distances between rDNA ITS-2 nucleotide sequences according to PAUP, including the** ***Lymnaea schirazensis*** **sequences obtained, together with species of the** ***Galba*** **/** ***Fossaria*** **group and selected species representing stagnicolines and** ***Pseudosuccinea*** **available in GenBank.** Below diagonal = total character differences; above diagonal = mean character differences (adjusted for missing data).
(PDF)

Table S3 **Pairwise distances between rDNA ITS-1 nucleotide sequences according to PAUP, including the** ***Lymnaea schirazensis*** **sequences obtained, together with species of the** ***Galba*** **/** ***Fossaria*** **group and selected species representing stagnicolines and** ***Pseudosuccinea*** **available in GenBank.** Below diagonal = total character differences; above diagonal = mean character differences (adjusted for missing data).
(PDF)

Table S4 **Pairwise distances between mtDNA** ***cox*** **1 nucleotide sequences according to PAUP, including the lymnaeid species studied, together with species of the** ***Galba*** **/** ***Fossaria*** **group and other proximal lymnaeid species available in GenBank (only** ***cox*** **1 sequence fragments of a length similar to that of sequences obtained in present paper).** Below diagonal = total character differences; above diagonal = mean character differences (adjusted for missing data). Haplotype codes only provisional due to incomplete sequences of the gene.
(PDF)

Table S5 **Lymnaeid shell measurement comparison between different experimentally-maintained populations of** ***Lymnaea schirazensis*** **from different geographical origins of Mexico.** Range include minimum and maximum extremes, with mean±standard deviation SD in parentheses. Measurements in mm. n = number of specimens measured.
(PDF)
