# Supplementary figures and images for: Lymnaea schirazensis, an Overlooked Snail Distorting Fascioliasis Data: Genotype, Phenotype, Ecology, Worldwide Spread, Susceptibility, Applicability
Source: PLoS One. 2011 Sep 29;6(9):e24567. doi: 10.1371/journal.pone.0024567 (PMC3183092; doi:10.1371/journal.pone.0024567)

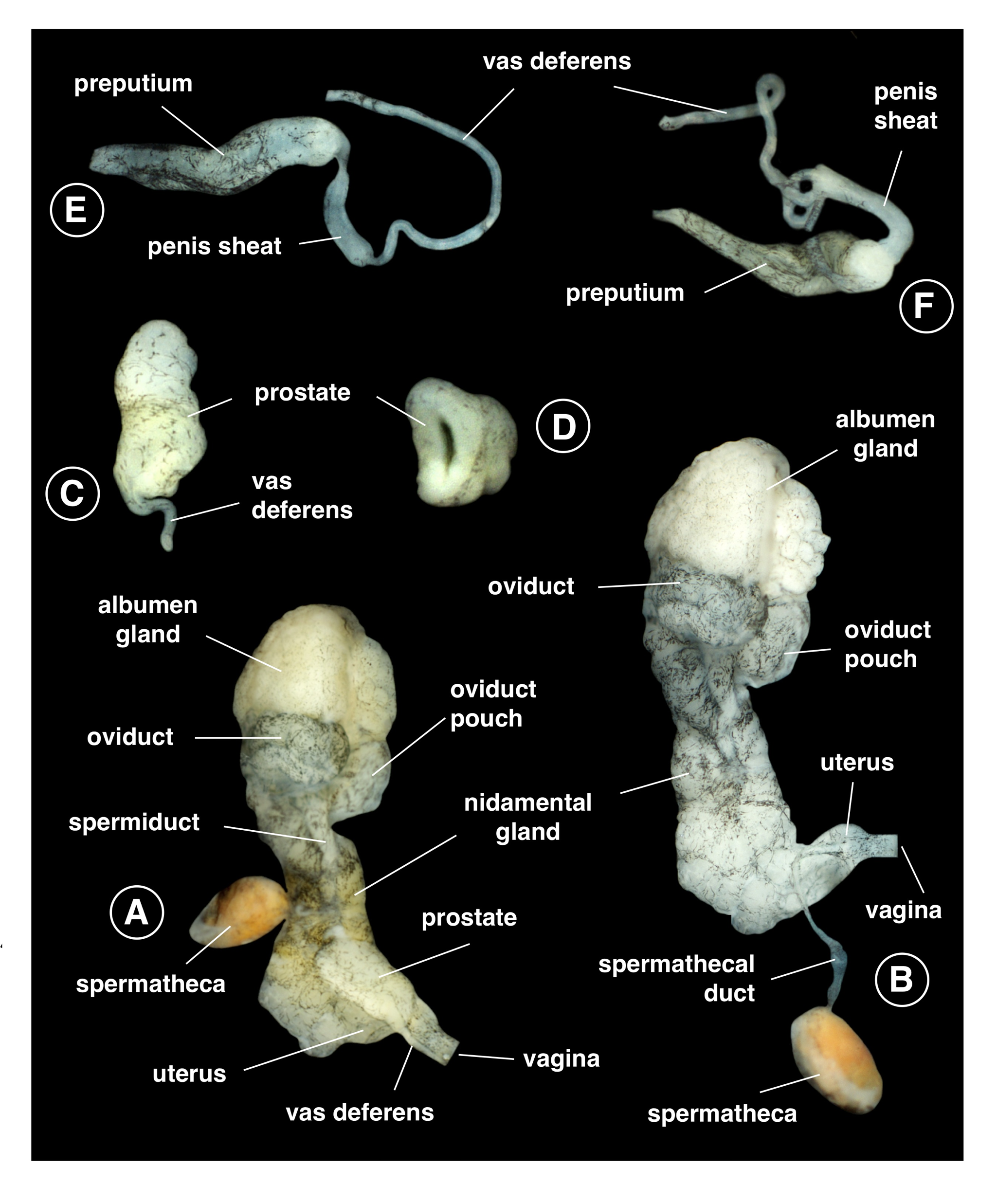

Supplement: Figure S1 — Photographs showing soft parts of Lymnaea schirazensis: A, B) part of reproductive system in ventral view (prostate removed in B); C) prostate and beginning of vas deferens; D) section of prostate showing absence of internal folds; E, F) male terminal organs. (TIFF) [file pone.0024567.s001.tiff]

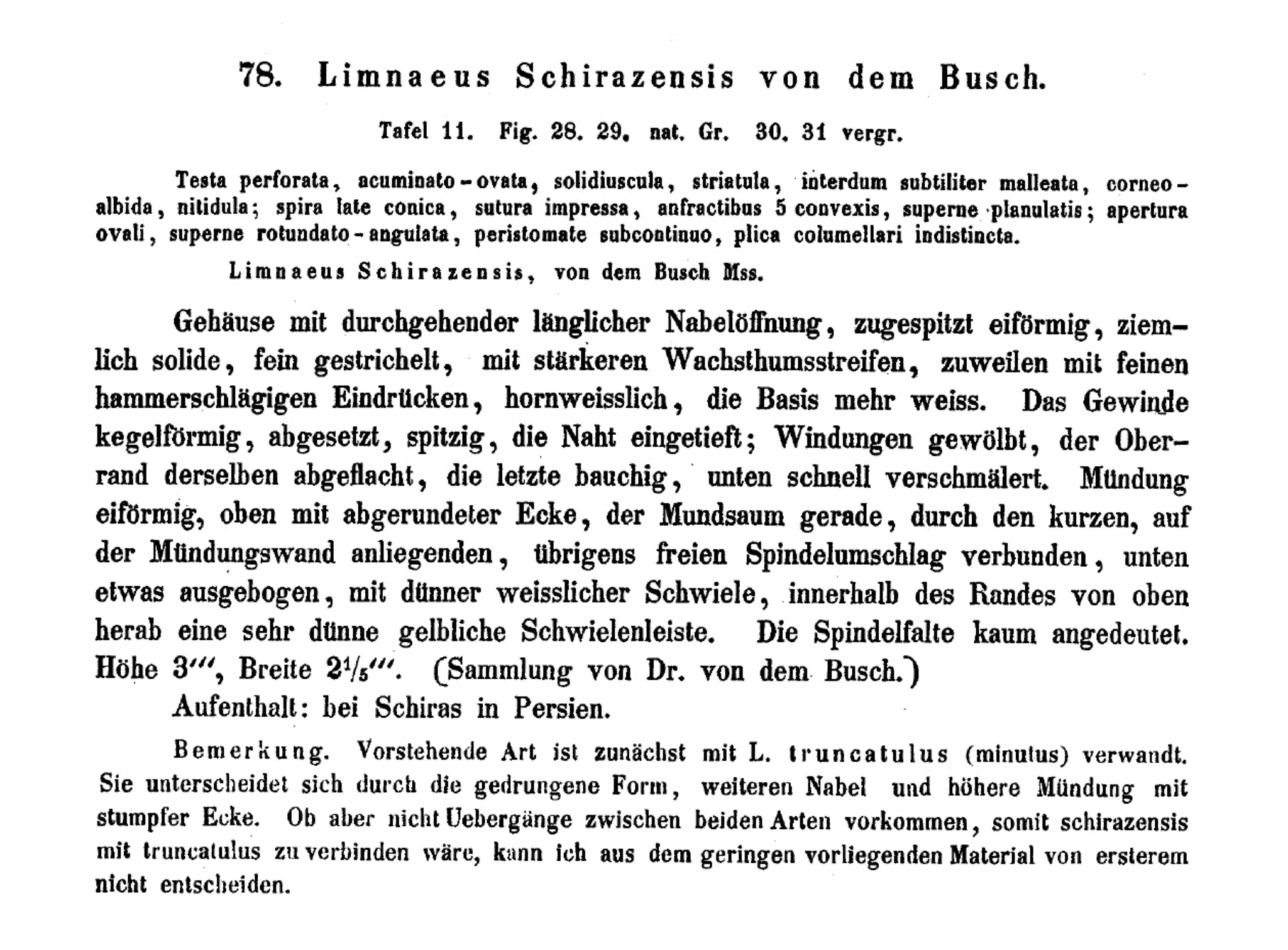

Supplement: Figure S2 — Original description of Lymnaea schirazensis by Küster in 1863 [71] according to snail materials collected by von dem Busch in the locality of Shiraz, Iran (described under species number 78, page 53, issue 184, year 1863). (TIFF) [file pone.0024567.s002.tiff]

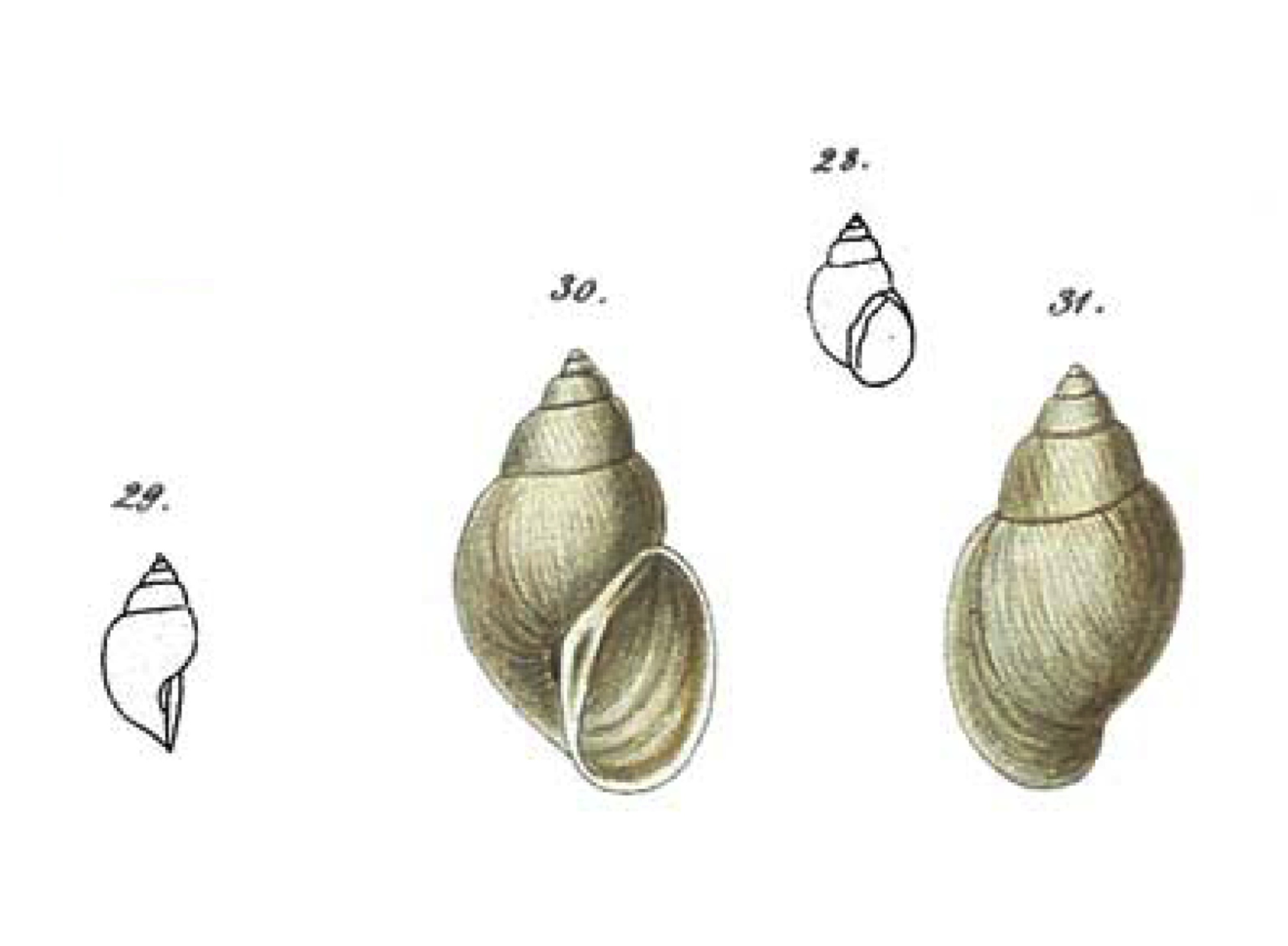

Supplement: Figure S3 — Original figures of Lymnaea schirazensis by Küster in 1862 [71] according to snail materials collected by von dem Busch in the locality of Shiraz, Iran (drawings in plate 11: figure numbers 28 and 29 showing natural size specimens and figures 30 and 31 showing enlarged specimen, in issue 182, year 1962). (TIFF) [file pone.0024567.s003.tiff]
