# Supplementary material for: Lymnaea schirazensis, an Overlooked Snail Distorting Fascioliasis Data: Genotype, Phenotype, Ecology, Worldwide Spread, Susceptibility, Applicability
Source: PLoS One. 2011 Sep 29;6(9):e24567. doi: 10.1371/journal.pone.0024567 (PMC3183092; doi:10.1371/journal.pone.0024567)
Supplement: Table S1 — List of species (and subspecies) of molluscs and trematode parasites included in this study according to the zoological nomenclature, in alphabetical order according to names used in the text. Clarification notes in brackets; s.l. = sensu lato. (PDF) [file pone.0024567.s004.pdf]

Table S1. List of species (and subspecies) of molluscs and trematode parasites included in this study according to the zoological nomenclature, in alphabetical order according to names used in the text. Clarification notes in parentheses; *s.l.* = *sensu lato*.

LYMNAEIDAE SPECIES:

— *Austropeplea tomentosa* (L. Pfeiffer, 1855)
— *Catascopia catascopium* (Say, 1817)
— *Catascopia elodes* (Say, 1821) (= *Stagnicola elodes* auctt.)
— *Catascopia emarginata* (Say, 1821)
— *Catascopia occulta* (Jackiewicz, 1959) (the synonymy with *Limnaea palustris* var. *terebra* Westerlund, 1885 has recently been proposed, although it is still pending molecular confirmation –- see: Vinarski MV, Glöer P (2008) Taxonomic notes on Euro-Siberian freshwater molluscs. 3. *Galba occulta* Jackiewicz, 1959 is a junior synonym of *Limnaea palustris* var. *terebra* Westerlund, 1885. Mollusca 26, 2: 175-185)
— *Galba pusilla* Schrank, 1803
— *Galba truncatula* (O.F. Müller, 1774)
— *Galba truncatula* variety *lanceolata* (Bourguignat, 1864)
— *Galba truncatula* variety *major* (Bourguignat, 1864)
— *Galba truncatula* variety *minutissima* (Bourguignat, 1864)
— *Galba truncatula* variety *neapolitana* (Bellini, 1904)
— *Galba truncatula* variety *submalleata* (Bourguignat, 1864)
— *Galba truncatula* variety *telouetensis* (Pallary, 1922)
— *Galba truncatula* variety *thiesseae* (Clessin, 1879)
— *Hinkleyia caperata* (Say, 1829)
— *Lymnaea (Lymnaea) stagnalis* (Linnaeus, 1758
— *Lymnaea (Stagnicola) fuscus* (C. Pfeiffer, 1821) (genus/subgenus status of *Stagnicola* still under discussion)
— *Lymnaea (Stagnicola) palustris* (O.F. Müller, 1774) (genus/subgenus status of *Stagnicola* still under discussion)
— *Lymnaea (Stagnicola) palustris palustris* (O.F. Müller, 1774) (genus/subgenus status of *Stagnicola* still under discussion)
— *Lymnaea (Stagnicola) palustris turricula* (Held, 1836) (species/subspecies status of *turricula* still under discussion; genus/subgenus status of *Stagnicola* still under discussion)
— *Lymnaea s.l. bulimoides* Lea, 1841 (= *Fossaria bulimoides* auctt.)
— *Lymnaea s.l. cousini* Jousseaume, 1887
— *Lymnaea s.l. cubensis* L. Pfeiffer, 1839
— *Lymnaea s.l. delaunayi* Folin, 1878
— *Lymnaea s.l. diaphana* King et Broderip, 1830 (authorship of the original publication is indicated as: P.P. King, assisted by W.J. Broderip; hence, should be better translated as King et Broderip, as already mentioned in Sherborn Index Animalium: Zoological Journal, Part 19, vol. V, July 1832)
— *Lymnaea s.l. hordeum* Mousson, 1874
— *Lymnaea s.l. humilis* Say, 1822
— *Lymnaea s.l. meridensis* Bargues, Artigas, Khoubbane et Mas-Coma, 2011
— *Lymnaea s.l. modicella* Say, 1825
— *Lymnaea s.l. neotropica* Bargues, Artigas, Mera y Sierra, Pointier et Mas-Coma, 2007
— *Lymnaea s.l. obrussa* Say, 1825 (= *Fossaria obrussa* auctt.)
— *Lymnaea s.l.* persica G.B. Sowerby II, 1872
— *Lymnaea s.l. schirazensis* Küster, 1862 (in [71], although text description was in 1863 and figure description in 1862, the year 1862 prevails because the name was already correctly cited in the figure legends of the 1862 plate – see article 12.2.7 of the International Code of Zoological Nomenclature)
— *Lymnaea s.l. viatrix* d'Orbigny, 1835 (= feminine spelling of original *Limnaeus viator*; see: Paraense WL (1976) *Lymnaea viatrix*: a study of topotypic specimens (Mollusca: Lymnaeidae). Rev Brasil Biol 36: 419-428; according to articles 31.2.1 and 34.2.1 of the International Code of Zoological Nomenclature, correct species name is *viator*, even when using the feminine genus *Lymnaea* Lamarck, 1799; however, the present paper does not appear to be the appropriate

place to reintroduce such a correction in the literature and thus the spelling *viatrix* used by everybody in recent decades is herein also used throughout)

— *Lymnaea s.l. zrmanjae* Brusina, 1866 (cited as possible synonym of *Galba truncatula* in [73]; ascription to genus erroneus; belongs to Hydrobiidae as verified by the same author somewhat later (Brusina, 1902 in [85]); correct ascription: *Tanousia zrmanjae* (Brusina, 1866))
— *Omphiscola glabra* (O.F. Müller, 1774)
— *Pseudosuccinea columella* (Say, 1817)
— *Radix auricularia* (Linnaeus, 1758)
— *Radix balthica* (Linnaeus, 1758)
— *Stagnicola bonnevillensis* (Call, 1884) (ascription to *Stagnicola* doubtful; should be momentaneously better included in *Lymnaea s.l.*)

PLANORBIDAE SPECIES:
— *Biomphalaria glabrata* (Say, 1818)
— *Biomphalaria pfeifferi* (Kraus, 1848)

TREMATODE SPECIES:
— *Fasciola gigantica* Cobbold, 1855
— *Fasciola hepatica* (Linnaeus, 1758)
— *Schistosoma mansoni* Sambon, 1907
