# Supplementary material for: Lymnaea schirazensis, an Overlooked Snail Distorting Fascioliasis Data: Genotype, Phenotype, Ecology, Worldwide Spread, Susceptibility, Applicability
Source: PLoS One. 2011 Sep 29;6(9):e24567. doi: 10.1371/journal.pone.0024567 (PMC3183092; doi:10.1371/journal.pone.0024567)
Supplement: Table S2 — Pairwise distances between rDNA ITS-2 nucleotide sequences according to PAUP, including the Lymnaea schirazensis sequences obtained, together with species of the Galba / Fossaria group and selected species representing stagnicolines and Pseudosuccinea available in GenBank. Below diagonal = total character differences; above diagonal = mean character differences (adjusted for missing data). (PDF) [file pone.0024567.s005.pdf]

Table S2. Pairwise distances between rDNA ITS-2 nucleotide sequences according to PAUP, including the *Lymnaea schirazensis* sequences obtained, together with species of the *Galba*/*Fossaria* group and selected species representing stagnicolines and *Pseudosuccinea* available in GenBank.

	1	2	3	4	5	6	7	8	9	10	11	12	13	14	15
1	-	0.22359	0.25714	0.30118	0.36408	0.32425	0.26633	0.26382	0.26633	0.34699	0.34521	0.41822	0.38095	0.36461	0.27390
2	91	-	0.06971	0.26256	0.33573	0.30133	0.19895	0.19634	0.19895	0.32955	0.33048	0.39320	0.34444	0.32164	0.21374
3	108	29	-	0.25522	0.33413	0.29491	0.21485	0.21220	0.21751	0.30986	0.30986	0.38889	0.32044	0.29885	0.23018
4	128	115	110	-	0.16143	0.14044	0.21316	0.21053	0.21053	0.33915	0.33835	0.42489	0.36779	0.34805	0.24359
5	150	140	139	77	-	0.03373	0.27322	0.27049	0.27322	0.32500	0.32581	0.39243	0.37379	0.34921	0.29443
6	119	113	110	58	14	-	0.21807	0.21495	0.21807	0.31759	0.31842	0.36388	0.34625	0.32410	0.26765
7	106	76	81	81	100	70	-	0.00249	0.00249	0.21782	0.21523	0.33957	0.28144	0.25000	0.26171
8	105	75	80	80	99	69	1	-	0.00499	0.21452	0.21192	0.33690	0.27844	0.24684	0.25895
9	106	76	82	80	100	70	1	2	-	0.21782	0.21523	0.33957	0.28144	0.25000	0.26171
10	127	116	110	136	130	121	66	65	66	-	0.00000	0.28645	0.27378	0.24752	0.31269
11	126	116	110	135	130	121	65	64	65	0	-	0.27792	0.26241	0.24566	0.31269
12	179	162	161	198	166	135	127	126	127	112	107	-	0.14532	0.14213	0.40751
13	152	124	116	153	154	134	94	93	94	118	111	59	-	0.03063	0.36145
14	136	110	104	134	132	117	79	78	79	100	99	56	14	-	0.34277
15	106	84	90	95	111	91	95	94	95	101	101	152	120	109	-

Below diagonal = total character differences; above diagonal = mean character differences (adjusted for missing data). Sequence correspondences: 1 = *L.* (*S.*) *p. palustris* H1 from Denmark [39]; 2 = *C. occulta* H1 from Poland [39]; 3 = *C. catascopium* from USA [38]; 4 = *L. cubensis* H1 from Cuba [15]; 5 = *L. viatrix* H1 from Argentina [15]; 6 = *L. neotropica* H1 from Peru [15]; 7 = *G. truncatula* H1 from Europe [15]; 8 = *G. truncatula* H2 from Morocco [15]; 9 = *G. truncatula* H3 from Bolivia [15]; 10 = *L. schirazensis* H1 (present paper); 11 = *L. schirazensis* H2 (present paper); 12 = *L. humilis* H1 from USA [45]; 13 = *L. cousini* H1 from Ecuador [55]; 14 = *L. meridensis* H1 from Venezuela [55]; 15 = *P. columella* H1 from Puerto Rico [55].
