# Supplementary material for: Lymnaea schirazensis, an Overlooked Snail Distorting Fascioliasis Data: Genotype, Phenotype, Ecology, Worldwide Spread, Susceptibility, Applicability
Source: PLoS One. 2011 Sep 29;6(9):e24567. doi: 10.1371/journal.pone.0024567 (PMC3183092; doi:10.1371/journal.pone.0024567)
Supplement: Table S3 — Pairwise distances between rDNA ITS-1 nucleotide sequences according to PAUP, including the Lymnaea schirazensis sequences obtained, together with species of the Galba / Fossaria group and selected species representing stagnicolines and Pseudosuccinea available in GenBank. Below diagonal = total character differences; above diagonal = mean character differences (adjusted for missing data). (PDF) [file pone.0024567.s006.pdf]

Table S3. Pairwise distances between rDNA ITS-1 nucleotide sequences according to PAUP, including the *Lymnaea schirazensis* sequences obtained, together with species of the *Galba*/*Fossaria* group and selected species representing stagnicolines and *Pseudosuccinea* available in GenBank.

	1	2	3	4	5	6	7	8	9	10	11	12	13	14	15
1	-	0.26476	0.27237	0.28958	0.28884	0.30816	0.26360	0.26151	0.26360	0.28571	0.28274	0.32727	0.29184	0.29652	0.33613
2	139	-	0.08068	0.27879	0.27606	0.29901	0.20816	0.21020	0.21020	0.28543	0.28457	0.29550	0.30000	0.29762	0.32922
3	140	43	-	0.28542	0.28429	0.31020	0.22727	0.22934	0.22934	0.27536	0.27443	0.28427	0.29839	0.29388	0.33755
4	139	138	137	-	0.07648	0.06312	0.15106	0.15319	0.15532	0.16896	0.16371	0.19000	0.20898	0.19200	0.28266
5	145	143	143	40	-	0.09560	0.15524	0.15726	0.15927	0.16893	0.16569	0.20157	0.19574	0.20623	0.28049
6	151	151	152	32	50	-	0.19462	0.19669	0.19876	0.18474	0.17944	0.20363	0.20240	0.20800	0.28306
7	126	102	110	71	77	94	-	0.00198	0.00397	0.17410	0.17271	0.20417	0.18644	0.19665	0.27426
8	125	103	111	72	78	95	1	-	0.00595	0.17622	0.17484	0.20625	0.18856	0.19874	0.27215
9	126	103	111	73	79	96	2	3	-	0.17834	0.17697	0.20833	0.19068	0.20084	0.27215
10	138	143	133	86	87	92	82	83	84	-	0.00188	0.17969	0.17115	0.15686	0.27719
11	136	142	132	83	85	89	81	82	83	1	-	0.17451	0.16795	0.15157	0.27623
12	162	151	141	95	103	101	98	99	100	92	89	-	0.13187	0.12200	0.28481
13	143	153	148	107	101	101	88	89	90	89	87	72	-	0.02698	0.28270
14	145	150	144	96	106	104	94	95	96	80	77	66	15	-	0.29724
15	160	160	160	132	138	137	130	129	129	130	129	135	134	140	-

Below diagonal = total character differences; above diagonal = mean character differences (adjusted for missing data). Sequence correspondences: 1 = *L.* (*S.*) *p. palustris* HA from Denmark [39]; 2 = *C. occulta* HA from Poland [39]; 3 = *C. catascopium* from USA [38]; 4 = *L. cubensis* HA from Cuba [15]; 5 = *L. viatrix* HA from Argentina [15]; 6 = *L. neotropica* HA from Peru [15]; 7 = *G. truncatula* HA from Europe [15]; 8 = *G. truncatula* HB from Morocco [15]; 9 = *G. truncatula* HC from Bolivia [15]; 10 = *L. schirazensis* HA (present paper); 11 = *L. schirazensis* HB (present paper); 12 = *L. humilis* HA from USA [45]; 13 = *L. cousini* HA from Ecuador [55]; 14 = *L. meridensis* HA from Venezuela [55]; 15 = *P. columella* HA from Puerto Rico [55].
