# Supplementary material for: Lymnaea schirazensis, an Overlooked Snail Distorting Fascioliasis Data: Genotype, Phenotype, Ecology, Worldwide Spread, Susceptibility, Applicability
Source: PLoS One. 2011 Sep 29;6(9):e24567. doi: 10.1371/journal.pone.0024567 (PMC3183092; doi:10.1371/journal.pone.0024567)
Supplement: Table S4 — Pairwise distances between mtDNA cox 1 nucleotide sequences according to PAUP, including the lymnaeid species studied, together with species of the Galba / Fossaria group and other proximal lymnaeid species available in GenBank (only cox 1 sequence fragments of a length similar to that of sequences obtained in present paper). Below diagonal = total character differences; above diagonal = mean character differences (adjusted for missing data). Haplotype codes only provisional due to incomplete sequences of the gene. (PDF) [file pone.0024567.s007.pdf]

Table S4. Pairwise distances between mtDNA *cox*1 nucleotide sequences according to PAUP, including the lymnaeid species studied, together with species of the *Galba*/*Fossaria* group and other proximal lymnaeid species available in GenBank.

	1	2	3	4	5	6	7	8	9	10	11	12	13	14	15	16	17	18	19	20	21
1	–	0.00149	0.05655	0.02083	0.02232	0.03443	0.11161	0.12000	0.11458	0.11458	0.11458	0.12054	0.10714	0.10714	0.10565	0.11458	0.11458	0.11310	0.10268	0.14583	0.15123
2	1	–	0.05804	0.01935	0.02083	0.03286	0.11012	0.11833	0.11310	0.11310	0.11310	0.11905	0.10565	0.10565	0.10417	0.11310	0.11310	0.11161	0.10119	0.14435	0.14969
3	38	39	–	0.04315	0.04464	0.05008	0.10119	0.10500	0.10417	0.10119	0.10119	0.10417	0.10714	0.11012	0.11161	0.10565	0.10565	0.10417	0.09375	0.14881	0.15278
4	14	13	29	–	0.00149	0.02191	0.09970	0.10667	0.10863	0.10565	0.10565	0.10863	0.10119	0.09821	0.09970	0.10119	0.10119	0.09970	0.09226	0.14435	0.14969
5	15	14	30	1	–	0.02347	0.10119	0.10833	0.11012	0.10714	0.10714	0.11012	0.10268	0.09970	0.10119	0.10268	0.10268	0.10119	0.09375	0.14583	0.15123
6	22	21	32	14	15	–	0.10798	0.10829	0.11111	0.10798	0.10798	0.11111	0.10955	0.10955	0.11111	0.11111	0.11111	0.10955	0.10329	0.14867	0.14867
7	75	74	68	67	68	69	–	0.00333	0.09673	0.09375	0.09226	0.09970	0.08929	0.08780	0.08929	0.09226	0.09226	0.09077	0.09375	0.14286	0.14815
8	72	71	63	64	65	64	2	–	0.09833	0.09500	0.09500	0.10167	0.09667	0.09500	0.09667	0.09833	0.09667	0.09500	0.10000	0.15000	0.15000
9	77	76	70	73	74	71	65	59	–	0.00595	0.00893	0.00744	0.08929	0.09375	0.09226	0.09226	0.09375	0.09226	0.08780	0.12500	0.12963
10	77	76	68	71	72	69	63	57	4	–	0.00595	0.01042	0.09077	0.09226	0.09375	0.08929	0.09077	0.08929	0.08482	0.12500	0.12963
11	77	76	68	71	72	69	62	57	6	4	–	0.01339	0.08929	0.09077	0.09226	0.08631	0.08780	0.08631	0.08185	0.11905	0.12346
12	81	80	70	73	74	71	67	61	5	7	9	–	0.09524	0.09673	0.09821	0.09673	0.09821	0.09673	0.08631	0.12649	0.13117
13	72	71	72	68	69	70	60	58	60	61	60	64	–	0.00744	0.00595	0.05804	0.05804	0.05655	0.05804	0.13542	0.14043
14	72	71	74	66	67	70	59	57	63	62	61	65	5	–	0.00149	0.05655	0.05655	0.05506	0.05952	0.13542	0.14043
15	71	70	75	67	68	71	60	58	62	63	62	66	4	1	–	0.05804	0.05804	0.05655	0.06101	0.13690	0.14198
16	77	76	71	68	69	71	62	59	62	60	58	65	39	38	39	–	0.00298	0.00446	0.05506	0.13542	0.14043
17	77	76	71	68	69	71	62	58	63	61	59	66	39	38	39	2	–	0.00149	0.05655	0.13690	0.14198
18	76	75	70	67	68	70	61	57	62	60	58	65	38	37	38	3	1	–	0.05804	0.13542	0.14043
19	69	68	63	62	63	66	63	60	59	57	55	58	39	40	41	37	38	39	–	0.13393	0.13889
20	98	97	100	97	98	95	96	90	84	84	80	85	91	91	92	91	92	91	90	–	0.00000
21	98	97	99	97	98	95	96	90	84	84	80	85	91	91	92	91	92	91	90	0	–

Only *cox*1 sequence fragments of a length similar to that of sequences obtained in present paper are included. Below diagonal = total character differences; above diagonal = mean character differences (adjusted for missing data). Haplotype codes only provisional due to incomplete sequences of the gene. Sequence correspondences: 1 = *L. cubensis cox*1-a from Cuba [15]; 2 = *L. cubensis cox*1-b from USA [45]; 3 = *L. viatrix cox*1-a from Argentina [15]; 4 = *L. neotropica cox*1-a from Peru [15]; 5 = *L. neotropica cox*1-b from Argentina [58]; 6 = *F. bulimoides* from USA, without provisional code ascription due to undetermined nucleotides in the sequence [44]; 7 = *G. truncatula cox*1-a from Spain [15]; 8 = *G. truncatula* from Germany, without provisional code ascription due to undetermined nucleotides in the sequence [57]; 9 = *L. schirazensis cox*1-a from Iran, Egypt, Spain, Dominican Republic, Mexico, Venezuela, Ecuador and Peru (present paper); 10 = *L. schirazensis cox*1-b from Mexico (present paper); 11 = *L. schirazensis cox*1-c from Ecuador (present paper); 12 = *L. schirazensis cox*1-d from Peru (present paper); 13 = *L. humilis cox*1-a from USA [45]; 14 = *L. humilis cox*1-b from USA [45]; 15 = *L. humilis cox*1-c from USA [45]; 16 = *L. cousini cox*1-a from Ecuador [55]; 17 = *L. cousini cox*1-b from Colombia [55]; 18 = *L. cousini cox*1-c from Colombia [55]; 19 = *L. meridensis cox*1-a from Venezuela [55]; 20 = *P. columella cox*1-a from Puerto Rico [55]; 21 = *P. columella* from Australia, without provisional code ascription due to the shorter sequence fragment [44].
