# Supplementary material for: Lymnaea schirazensis, an Overlooked Snail Distorting Fascioliasis Data: Genotype, Phenotype, Ecology, Worldwide Spread, Susceptibility, Applicability
Source: PLoS One. 2011 Sep 29;6(9):e24567. doi: 10.1371/journal.pone.0024567 (PMC3183092; doi:10.1371/journal.pone.0024567)
Supplement: Table S5 — Lymnaeid shell measurement comparison between different experimentally-maintained populations of Lymnaea schirazensis from different geographical origins of Mexico. Range include minimum and maximum extremes, with mean±standard deviation SD in parentheses. Measurements in mm. n = number of specimens measured. (PDF) [file pone.0024567.s008.pdf]

Table S5. Lymnaeid shell measurement comparison between different experimentally-maintained populations of *Lymnaea schirazensis* from different geographical origins of Mexico.

Shell parameters (abbreviation)	Height (SH)	Maximum width (SW)	Aperture length (AL)	Aperture width (AW)	Last spire length (LSL)	Spiral angle (SSA)	Whorl number	SH/SW ratio	SH/AL ratio	SH/LSL ratio
Jiutepec, Morelos, Huauchinango (n = 30)	4.41-7.23 (5.21 ± 0.84)	2.43-3.81 (2.87 ± 0.43)	1.91-3.16 (2.34 ± 0.36)	1.27-2.00 (1.56 ± 0.24)	3.24-5.29 (3.90 ± 0.61)	33.13-51.52 (42.60 ± 3.88)	4-5 (4.07 ± 0.25)	1.59-1.99 (1.82 ± 0.08)	2.04-2.52 (2.23 ± 0.12)	1.26-1.40 (1.34 ± 0.03)
Escuela Obregon, Trinidad Tepango (n = 30)	4.95-6.29 (5.59 ± 0.35)	2.80-3.56 (3.11 ± 0.21)	2.53-3.15 (2.84 ± 0.20)	1.53-2.16 (1.83 ± 0.14)	3.94-5.16 (4.48 ± 0.29)	39.67-64.59 (47.61 ± 5.17)	4 (4.00 ± 0.00)	1.64-1.94 (1.80 ± 0.08)	1.78-2.23 (1.98 ± 0.10)	1.21-1.31 (1.25 ± 0.02)
Xalpatlaco population 1 (n = 30)	4.34-7.30 (5.88 ± 0.76)	2.12-3.99 (3.14 ± 0.48)	1.88-3.13 (2.61 ± 0.35)	1.27-2.44 (1.81 ± 0.30)	3.17-5.19 (4.37 ± 0.54)	26.48-53.49 (41.76 ± 7.03)	4-5 (4.30 ± 0.47)	1.66-2.05 (1.88 ± 0.10)	1.96-2.62 (2.26 ± 0.14)	1.22-1.44 (1.35 ± 0.05)
Xalpatlaco population 2 (n = 30)	3.27-5.86 (4.43 ± 0.65)	1.82-3.16 (2.48 ± 0.31)	1.51-2.95 (2.15 ± 0.32)	1.06-1,72 (1.39 ± 0.16)	2.57-4.59 (3.42 ± 0.49)	28.22-48.47 (38.94 ± 5.22)	3-4 (3.97 ± 0.18)	1.58-1.95 (1.78 ± 0.09)	1.84-2.29 (2.07 ± 0.11)	1.23-1.36 (1.30 ± 0.03)
Atlixco, Puebla (n = 14)	3.73-7.25 (5.05 ± 0.94)	2.34-3.82 (2.82 ± 0.43)	1.85-3.34 (2.38 ± 0.44)	1.29-2.02 (1.61 ± 0.25)	2.97-5.47 (3.87 ± 0.73)	31.08-60.36 (43.94 ± 7.17)	4-5 (4.07 ± 0.27)	1.60-1.93 (1.79 ± 0.10)	1.93-2.25 (2.13 ± 0.09)	1.24-1.35 (1.31 ± 0.03)
TOTAL (n = 134)	3.27-7.30 (5.26 ± 0.87)	1.82-3.99 (2.89 ± 0.45)	1.51-3.34 (2.47 ± 0.41)	1.06-2.44 (1.64 ± 0.28)	2.57-5.47 (4.02 ± 0.65)	26.48-64.59 (42.85 ± 6.31)	3-5 (4.08 ± 0.30)	1.58-2.05 (1.82 ± 0.09)	1.78-2.62 (2.13 ± 0.16)	1.21-1.44 (1.31 ± 0.05)

Range include minimum and maximum extremes, with mean ± standard deviation SD in parentheses. Measurements in mm. n = number of specimens measured.
